# Size Dependent Photocatalytic Activity of Mesoporous ZnIn_2_S_4_ Nanocrystal Networks

**DOI:** 10.1021/acscatal.4c04195

**Published:** 2024-09-12

**Authors:** Evangelos K. Andreou, Ioannis Vamvasakis, Andreas Douloumis, Georgios Kopidakis, Gerasimos S. Armatas

**Affiliations:** Department of Materials Science and Engineering, University of Crete, Heraklion 70013, Greece

**Keywords:** thiospinels, zinc indium sulfide, nanoporous materials, quantum confinement, hydrogen evolution

## Abstract

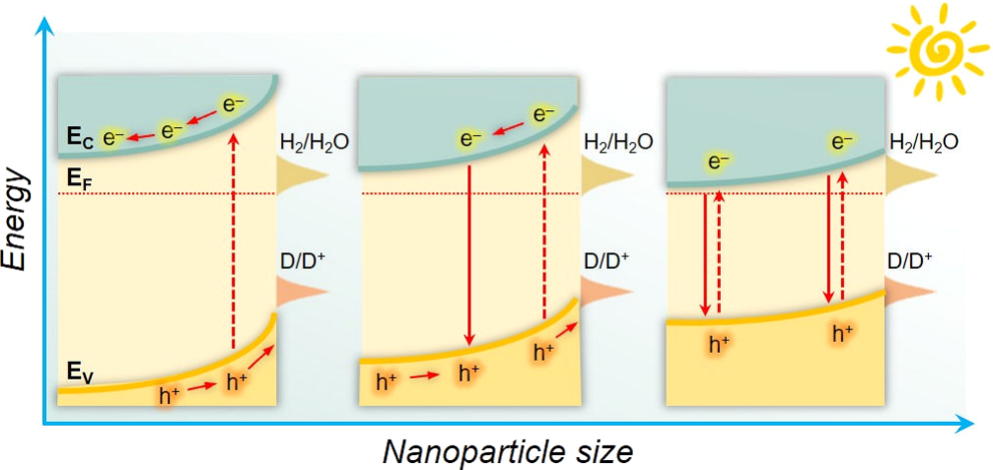

Understanding of the band-edge electronic structure and charge-transfer dynamics in size-confined nanostructures is vital in designing new materials for energy conversion applications, including green hydrogen production, decomposition of organic pollutants and solar cells. In this study, a series of mesoporous materials comprising continuous networks of linked zinc indium sulfide (ZnIn_2_S_4_) nanocrystals with a tunable diameter (ranging from 4 to 12 nm) is reported. These nanomaterials demonstrate intriguing size-dependent electronic properties, charge-transfer kinetics and photocatalytic behaviors. Our extensive characterizations uncover strong size effects on the catalytic activity of constituent ZnIn_2_S_4_ nanocrystals in the photochemical hydrogen evolution reaction. As an outcome, the optimized single-component ZnIn_2_S_4_ mesostructure produces hydrogen at a 7.8 mmol g_cat_^–1^ h^–1^ release rate under ultraviolet (UV)–visible light irradiation associated with an apparent quantum yield (AQY) of 17.2% at 420 ± 10 nm, far surpassing its microstructured counterpart by a factor of 10.7×. These findings provide a valuable perspective for the rational design of semiconductor nanostructures through synthetic engineering, aiming at the development of high-performance catalysts for zero-carbon energy-related applications.

## 1 Introduction

Semiconductor nanostructures are currently at the forefront of research in many solar energy conversion and photocatalytic technologies, such as photochemical water-splitting cells, photovoltaics and light-emitting devices. In principle, nanostructure engineering could enable unique functionalities such as size-dependent optical absorption and emission properties attributed to quantum confinement effects.^1^ Furthermore, reducing semiconductor particles to the nanoscale is highly desirable for light-induced catalysis due to the ensuing tunable photon-harvesting efficiency and high surface reactivity.^2^ So far, a diverse set of metal oxide (BiVO_4_, SrTiO_3_, Nb_2_O_5_, Cu_2_O, etc.), sulfide (MoS_2_, CdS, ZnS, etc.) and (oxy)nitride (Ta_2_N_5_, LaTiO_2_N, TaON, etc.) nanostructures with tailored compositions and morphologies have been explored as photocatalysts for water photosplitting reactions.^3−7^ To achieve high solar-to-hydrogen conversion efficiencies, research efforts are focusing on devising reliable semiconductor materials with rationally designed band-edge positions and catalytic activity.^8−10^ Among others, metal sulfides have emerged as well-performing candidates for various energy conversion systems, such as water electrolyzers, solar cells and supercapacitors. This is attributed to their large absorption coefficient (typically exceeding 10^4^ cm^–1^) in the visible region, prominent redox activity, and high carrier mobility (>100 cm^2^ V^–1^ s^–1^).^11,12^ Nevertheless, the photochemical efficiency of metal sulfide photocatalysts remains low and is fundamentally hindered by the poor chemical stability (being susceptible to anodic photocorrosion) and rapid recombination of photogenerated electron–hole pairs.

Recently, spinel chalcogenides (with a general formula of A^II^B^III^X_4_, in which A and B are a divalent and trivalent metal, respectively, and X = S, Se) have garnered significant interest for their potential in energy conversion and storage.^13−16^ The high visible-light harvesting ability, multiple redox behavior, excellent charge carriers’ mobility and remarkable photochemical stability have promoted their use as next-generation photocatalysts and solar energy collectors. Thus, over the last years, various high-performing thiospinel catalysts, including ZnIn_2_S_4_, NiCo_2_S_4_, and CuCo_2_S_4_, have been described for many photo- and electrochemical reactions, such as water electrolysis and hydrogen or oxygen evolution, CO_2_ and N_2_ fixation and Cr(VI) pollution remediation, showing promising results.^17−24^ Although encouraging, the photocatalytic activity of these materials, however, is often hindered by fast charge-carrier recombination and low density of surface-active sites.

We have recently demonstrated a low-temperature synthetic route to isolate fairly monodispersed thiospinel nanocrystals (NCs).^25,26^ In contrast to conventional solvothermal and high-temperature solid-state methods, this chemical process enables the synthesis of ultrasmall thiospinel nanoparticles with tunable size and composition. Such colloidal nanoparticles may constitute functional structural units to assemble three-dimensional (3D) mesostructured networks through a polymer-templating chemical method.^27^ In point of fact, mesoscopic architectures made from nanoscale building blocks can combine disparate functionalities within the same material, such as quantum-confined optical absorption and fast interparticle mass transport—capabilities not present in traditional nanoporous solids or isolated nanoparticles. Additionally, nanometer-scale structures can provide a notable boost in intrinsic photochemical activity by reducing bulk carrier recombination due to the shortened distance for charge carriers to reach the surface-active sites, a notorious problem in photocatalysis. Despite these advantages, a pertinent mechanistic scheme decoding the quantum confinement effects and reaction kinetics in these nanomaterials still remains elusive. In this work, we present the synthesis of new mesoporous frameworks consisting of ZnIn_2_S_4_ NCs with variable diameter (from ∼4 to ∼12 nm), which to our knowledge is the first example of porous thiospinel structures with tunable grain composition. Significantly, these ensemble nanostructures provide a unique opportunity for studying the size effect on the charge-transfer dynamics and catalytic properties of metal chalcogenide mesoporous architectures. This could enable investigation of whether quantum confinement of carriers affects the photochemical performance of nanoporous materials in a way similar to that observed in discrete nanoparticles. Through comprehensive physicochemical and (photo)electrochemical investigations as well as theoretical calculations, we gained an understanding of the interplay between size-dependent electronic structure (band-edge positions and charge density profile), interfacial charge transport and intrinsic photocatalytic behavior in this system. Together with optimized charge transport and separation kinetics within the NC-structure, the single-component ZnIn_2_S_4_ catalyst achieves a remarkable water photosplitting efficiency of up to 17.2% at 420 ± 10 nm accompanied by a H_2_ generation rate of 7.8 mmol g_cat_^–1^ h^–1^.

## 2 Results and Discussion

### 2.1 Synthesis, Structural Investigation, and Morphology

**Figure 1:**
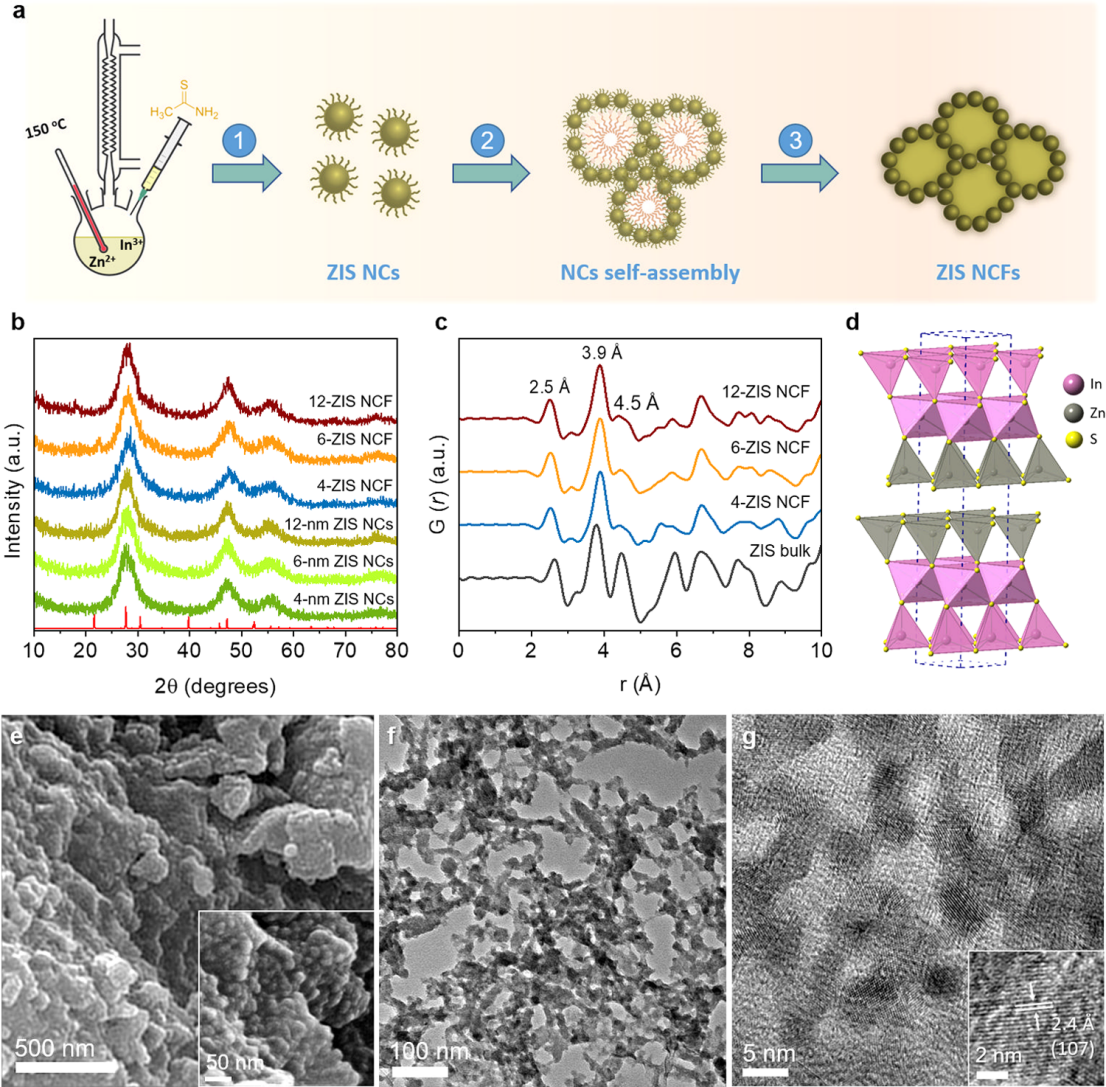
(a) Schematic illustration of the synthetic procedure of mesoporous ZIS nanocrystal frameworks (NCFs) (step I: reflux synthesis of 3-MPA-capped ZIS NCs; step II: polymer-assisted chemical self-assembly; step III: template extraction toward open-pore structures). (b) XRD patterns of the as-prepared ZIS NCs and mesoporous *n*-ZIS NCFs. The standard diffraction pattern of the hexagonal ZnIn_2_S_4_ (JCPDS card no. 65–2023; red line) is also given. (c) Reduced atomic pair distribution functions G(*r*) of the mesoporous *n*-ZIS NCFs and polycrystalline bulk ZIS. (d) Crystal structure of hexagonal ZnIn_2_S_4_ (space group: *P*3̅*m*1). Representative (e) FESEM, (f) TEM, and (g) HRTEM images of 6-ZIS NCF. The insets of panels (e, g) show an enlarged region of the images. a.u., arbitrary units.

An overview of the multistep synthesis process for the mesoporous ZnIn_2_S_4_ (ZIS) structures is shown in Figure 1a. Briefly, colloidal ZIS NCs with adjustable diameters were initially synthesized through a reflux reaction involving Zn and In nitrates (1:2 nominal ratio) and thioacetamide as precursors and ethylene glycol as the solvent. To control the crystal growth kinetics of ZIS and prevent the formation of large nanoparticle aggregates, we utilized 3-mercaptopropionic acid (3-MPA) as a surface capping agent. By varying the reaction time, we successfully prepared ZIS NCs with tunable particle sizes. The gradual growth of ZIS NCs was indicated by the color change of the isolated nanoparticles, transitioning from light to bright yellow. Then, taking advantage of the chemical self-assembly, we prepared different 3D mesostructured frameworks using these thiolate-capped ZIS NCs as secondary building blocks. This synthetic route involves H_2_O_2_-mediated oxidative coupling of colloidal ZIS NCs through the formation of S–S interparticle bonds around of block copolymer aggregates.^28^ Finally, the organic template was removed from the pores by dissolution in warm ethanol and water (∼40 °C) to give an extended mesoporous network structure with large accessible surface area and well-defined pores, denoted as *n*-ZIS NCFs (NCFs: NC-based frameworks), where *n* refers to the average size of constituent NCs. Thermogravimetric analysis (TGA) of the final products showed that approximately 9.7–12.2 wt % of organic content remains within the porous structure of *n*-ZIS NCFs (Figure S1, Supporting Information). Furthermore, the elemental composition of the prepared samples was analyzed with energy-dispersive X-ray spectroscopy (EDS). The EDS spectra obtained from multiple regions of samples reveal a S-defective structure for the 4-ZIS (Zn/In/S ratio ∼1.14:2:3.90) and a nearly stoichiometric composition (∼1:2:4 Zn/In/S ratio, within 5% deviation) for the 6-ZIS and 12-ZIS NCFs (Figure S2, Supporting Information); according to the EDS results, the percentage of sulfur-defects in 4-ZIS NCF is estimated to be ∼2.5%. The nonstoichiometric structure of 4-ZIS NCF likely results from the slower kinetics of In^3+^ ions in ethanediol compared to the smaller and more labile Zn^2+^ ions. It is widely recognized that unsaturated sulfur atoms may act as electron-trapping sites, which restrain electron–hole pair recombination and enhance photoabsorption. Additionally, such low-coordinated atoms may serve as highly active sites for proton capture, thereby expediting the kinetics of water reduction by lowering the overall activation barrier.^29−32^ On the other hand, defect midgap states induced by sulfur vacancies on the catalyst’s surface (interface gap states) may also act as recombination centers of photogenerated carriers, ultimately leading to a decrease in photocatalytic activity. For reference, polycrystalline bulk ZIS (denoted as ZIS bulk) has also been synthesized via a well-established hydrothermal method. This material exhibits an elemental composition of Zn/In/S close to a 1:2:4 ratio, as confirmed by EDS analysis. The analytic data and atomic contents of the prepared ZIS materials are listed in Table S1 in the Supporting Information. X-ray photoelectron spectroscopy (XPS) measurements were also performed to characterize the surface chemical states of the mesoporous materials. Coherent with the EDS results, the XPS survey spectra identify the coexistence of Zn, In, and S elements in the *n*-ZIS NCFs samples, while the detection of O signal can be attributed to the partial oxidation of surface atoms upon exposure to air (Figure S3a, Supporting Information). All the high-resolution Zn 2p XPS spectra show a doublet peak at 1024.4 and 1045.5 ± 0.1 eV binding energies, which correspond to the Zn 2p_3/2_ and 2p_1/2_ core-levels of divalent Zn ions, respectively (Figure S3b, Supporting Information).^33^ Similarly, the In 3d XPS spectra display a doublet peak with the In 3d_5/2_ and 3d_3/2_ spin–orbit states falling at 445.3 and 452.9 ± 0.1 eV, respectively, concerning the In–S coordination environment of In^3+^ in ZnIn_2_S_4_ (Figure S3c, Supporting Information).^34^ Meanwhile, a double deconvoluted peak at 162.0 and 163.3 ± 0.2 eV binding energies in the S 2p XPS spectra is consistent with the S 2p_3/2_ and 2p_1/2_ spin–orbits of S^2–^ state (Figure S3d, Supporting Information).^35^ Taken together, these results provide compelling evidence for the formation of ZnIn_2_S_4_ thiospinel structure.

The X-ray diffraction (XRD) patterns of the as-prepared 3-MPA-capped NCs and mesoporous *n*-ZIS NCFs samples are shown in Figure 1b. All the XRD plots display three broad diffraction peaks within the 20–60° 2θ scattering angles, attributable to the very small size of crystallites. The broadness of the XRD peaks makes it difficult to identify the crystal structure of the samples. To figure out the local atomic structure of the ZIS mesostructures, we conducted high-energy X-ray diffuse scattering (HE-XRDS) measurements and pair distribution function (PDF) analysis.^36^Figure 1c presents the PDF plots as a function of the interatomic distance for the mesoporous ZIS samples along with that of polycrystalline ZIS. The PDFs show interatomic vectors for the mesoporous ZIS materials that closely resemble those of the polycrystalline sample, testifying similar atomic configuration. In particular, the PDFs of *n*-ZIS NCFs appear consistent with the hexagonal crystal structure of ZnIn_2_S_4_ (space group: *P*3̅*m*1, Figure 1d), showing intense peaks at ∼2.5, ∼3.9, and ∼4.5 Å that correspond to the Zn/In–S first, Zn···Zn/In···In nearest and Zn···In next nearest neighbor distances, respectively, within the hexagonal ZnIn_2_S_4_. In agreement with this, the crystal structure of the polycrystalline sample obtained via the hydrothermal method was identified as hexagonal ZnIn_2_S_4_ (JCPDS card no. 65–2023) based on the XRD data (Figure S4, Supporting Information). Congruently, this analysis provides unequivocal evidence for the hexagonal thiospinel structure of the ZIS NCs. In addition, a more detailed look at the pair correlation peak for In···In and Zn···Zn second neighbors in mesoporous samples from 12-ZIS to 4-ZIS NCFs reveals a shift from 3.89 to 3.91 Å; in contrast, the position of the Zn/In–S correlation peak remains constant at 2.53 Å. These structural changes can be interpreted as slight distortions in the In–S–In/Zn–S–Zn bonds within the 4-ZIS lattice, likely stemming from the presence of sulfur vacancies, in line with EDS results (Figure S5, Supporting Information).

The morphology and crystal structure of *n*-ZIS NCFs were examined with field-emission scanning electron microscopy (FESEM) and transmission electron microscopy (TEM). Representative FESEM images in Figure 1e reveal that the 6-ZIS NCF sample has an open-up architecture consisting of fairly monodisperse nanoparticles with a size less than 10 nm. For comparison, FESEM observation over the reference polycrystalline ZIS shows individual micrometer-sized particles of ∼3–6 μm diameter, which are composed of plenty of intersecting nanosheets with a thickness of about 18–20 nm, see Figure S6 in the Supporting Information. Figure 1f–g displays typical TEM images obtained from mesoporous 6-ZIS NCF, while the TEM images for the other ZIS mesoporous are provided in Figure S7 in the Supporting Information. Direct TEM investigations disclose the formation of nanoporous networks composed of closely connected nanoparticles, which is beneficial to interparticle electron transfer. On the basis of TEM images, we obtained an average size of the constituent nanoparticles from ∼4 to ∼12.2 nm in the series of mesoporous *n*-ZIS NCFs materials, which is strongly related to the reaction time in the synthesis of starting NCs (see Figure S8, Supporting Information). These grain sizes are in excellent agreement with those obtained from independent small-angle X-ray scattering (SAXS) analysis; the diameter of constituent NCs in different ZIS mesoporous samples derived from the SAXS patterns ranges from ∼4.5 to ∼11.3 nm (Table 1 and Figure S9, Supporting Information). Moreover, the crystal structure of ZIS nanoparticles was further elucidated using high-resolution TEM (HRTEM). A closer analysis of the mesoporous structure in Figure 1g supports the hexagonal crystal phase of constituent NCs, in agreement with the PDF results, showing well-resolved lattice fringes with 2.4 Å *d*-spacing throughout the nanoparticles that correspond to the (107) planes of hexagonal ZnIn_2_S_4_ (JCPDS card no. 65–2023).

**Table 1:** Textural Parameters and Energy Bandgap of Mesoporous *n*-ZIS NCFs, ZIS RNAs, and Polycrystalline ZIS Materials

samples	surface area (m^2^ g^–1^)	pore volume^a^ (cm^3^ g^–1^)	pore size (nm)	*D*_TEM_ (*D*_SAXS_)^b^ (nm)	energy bandgap^c^ (eV)
4-ZIS NCF	207	0.17	6.4	4.0 ± 0.4 (4.5 ± 0.3)	2.75 (2.80)
6-ZIS NCF	195	0.17	6.4	6.5 ± 0.6 (6.2 ± 0.4)	2.66 (2.72)
12-ZIS NCF	187	0.16	6.3	12.2 ± 0.9 (11.3 ± 0.6)	2.65 (2.66)
ZIS RNAs	76	0.04	1.6		2.64
ZIS bulk	47	0.05		∼18–20 nm^d^	2.50

a Cumulative pore volume at relative pressure (*P*/*P*_0_) equal to 0.98.

b Average particle size and standard deviation of the constituent ZIS NCs estimated from TEM and SAXS (in parentheses) measurements.

c The energy gap obtained from the corresponding Tauc plots for indirect energy gap semiconductor. In parentheses: the energy bandgap of the precursor NCs.

d Wall thickness of intersecting nanosheets.

**Figure 2:**
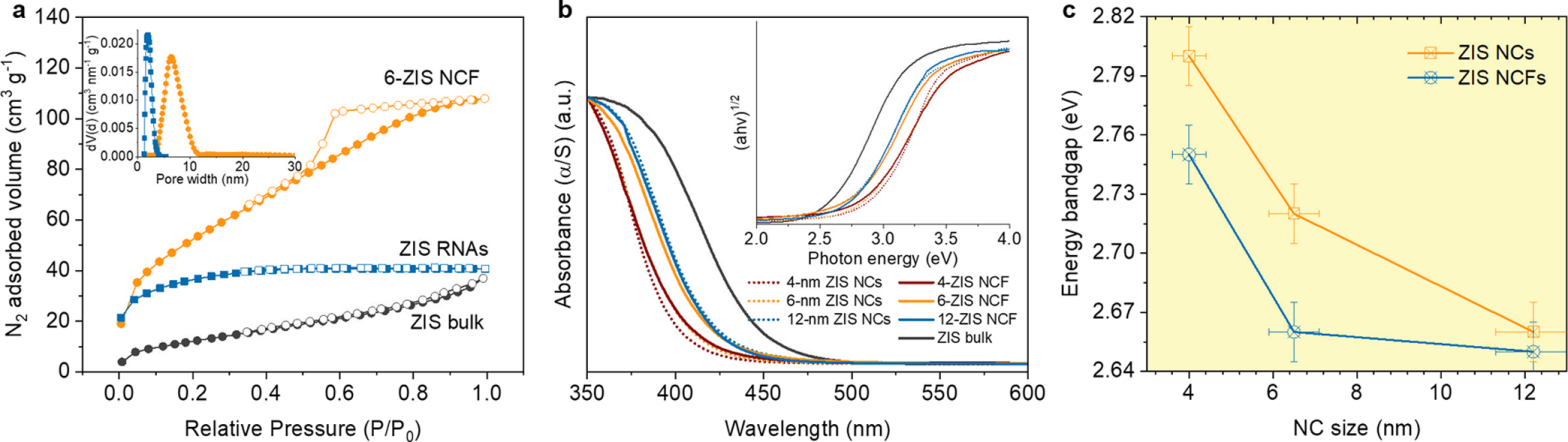
(a) N_2_ adsorption (filled symbols) and desorption (empty symbols) isotherms at −196 °C for the mesoporous 6-ZIS NCF, random ZIS NC-aggregates (ZIS RNAs) and polycrystalline ZIS. The isotherms of ZIS RNAs are shifted by 15 cm^3^ g^–1^ for clarity. Inset: the corresponding NLDFT pore-size distribution plots derived from the adsorption isotherms. (b) UV–vis absorption spectra and (inset) the corresponding Tauc plots of ZIS NCs, mesoporous *n*-ZIS NCFs and polycrystalline ZIS materials. (c) The energy bandgap as a function of the NC size for the as-prepared ZIS NCs and mesoporous *n*-ZIS NCFs. The size of constituent NCs was estimated from TEM analysis.

The porosity of the materials under study was determined using N_2_ physisorption measurements. As shown in Figures 2a and S10 in the Supporting Information, all *n*-ZIS NCFs samples feature typical type-IV N_2_ adsorption–desorption isotherms accompanied by an H_2_-type hysteresis loop, suggesting mesoporous solids with interconnected pores.^37^ These materials exhibited Brunauer–Emmett–Teller (BET) surface areas as high as 187–207 m^2^ g^–1^ and total pore volumes of 0.16–0.17 cm^3^ g^–1^. The small decrease in surface area results from the increased size of ZIS nanoparticles that compose the framework. However, all *n*-ZIS NCFs materials consistently maintain an open-pore structure with a large internal surface area. Comparatively, the bulk ZIS analog shows a considerably lower BET surface area of 47 m^2^ g^–1^. Since these mesoporous structures are derived as inorganic replicas from the same polymer template, the resulting *n*-ZIS NCFs exhibit very similar pore diameters. Analysis of the adsorption data using the nonlocal density functional theory (NLDFT) reveals quite narrow size distributions of pores with mesopore sizes ∼6.3–6.4 nm. This is the first example of mesoporous ZnIn_2_S_4_ materials with high internal surface area and well-defined pores. The advantage of polymer-templated synthesis was directly demonstrated through the comparative study of a ZIS RNAs reference material (RNAs: random NC-aggregates). This material was prepared via template-free oxidative coupling of 6 nm ZIS NCs that is expected to form dense assemblies of randomly agglomerated NCs. As illustrated in Figure 2a, ZIS RNAs exhibit a typical type-I adsorption isotherm, indicative of a microporous structure, with a calculated surface area of 76 m^2^ g^–1^ and a pore size of about 1.6 nm, which are notably lower than those of the templated cognate. The coalescence of ZIS nanoparticles is detrimental to catalysis, as it results in the formation of dense agglomerates with a limited number of exposed active sites. Table 1 lists the textural parameters of the different ZIS materials.

The ultraviolet–visible (UV–vis) diffuse reflectance spectra indicate that ZIS NCs exhibit a well-defined electronic structure. The UV–vis spectra in Figure 2b show sharp optical absorption onsets related to a systematic increase in the bandgap absorption from ∼2.66 to ∼2.80 eV with decreasing nanoparticle diameter from 12 to 4 nm as listed in Table 1. This shift of the energy gap is ascribed to size-induced quantum confinement transitions, similar to those observed in individual semiconductor quantum dots and clusters.^38^ The optical absorption edges of the mesoporous samples from 12-ZIS to 4-ZIS NCFs show a similar trend to the starting nanoparticles (from 2.65 to 2.75 eV), suggesting that quantization of the intrinsic band structure of the precursor NCs is well-preserved in the assembled structures, see Table 1 and Figure 2c. The small red-shift (∼50 meV) in the optical absorption going from colloidal NCs to mesoporous structures suggests strong electronic coupling and electron delocalization along the assembled frameworks, indicating a slight reduction in the quantum confinement effect. In comparison with the absorption spectrum of bulk ZIS (bandgap ∼2.50 eV), the *n*-ZIS NCFs series of materials experience a significantly higher bandgap absorption, which is attributed to the substantial size reduction of constituent NCs (ca. 4–12 nm in size, as inferred from SAXS and TEM results) that allows quantization of the band-edge electronic states. In line with its close-packed structure, the bandgap of the ZIS RNAs reference sample was measured to be ∼2.55 eV (Figure S11, Supporting Information), that is, lower than the bandgap of 6-ZIS NCF prepared by the polymer-templating method.

### 2.2 Photocatalytic Hydrogen Evolution Activity

**Figure 3:**
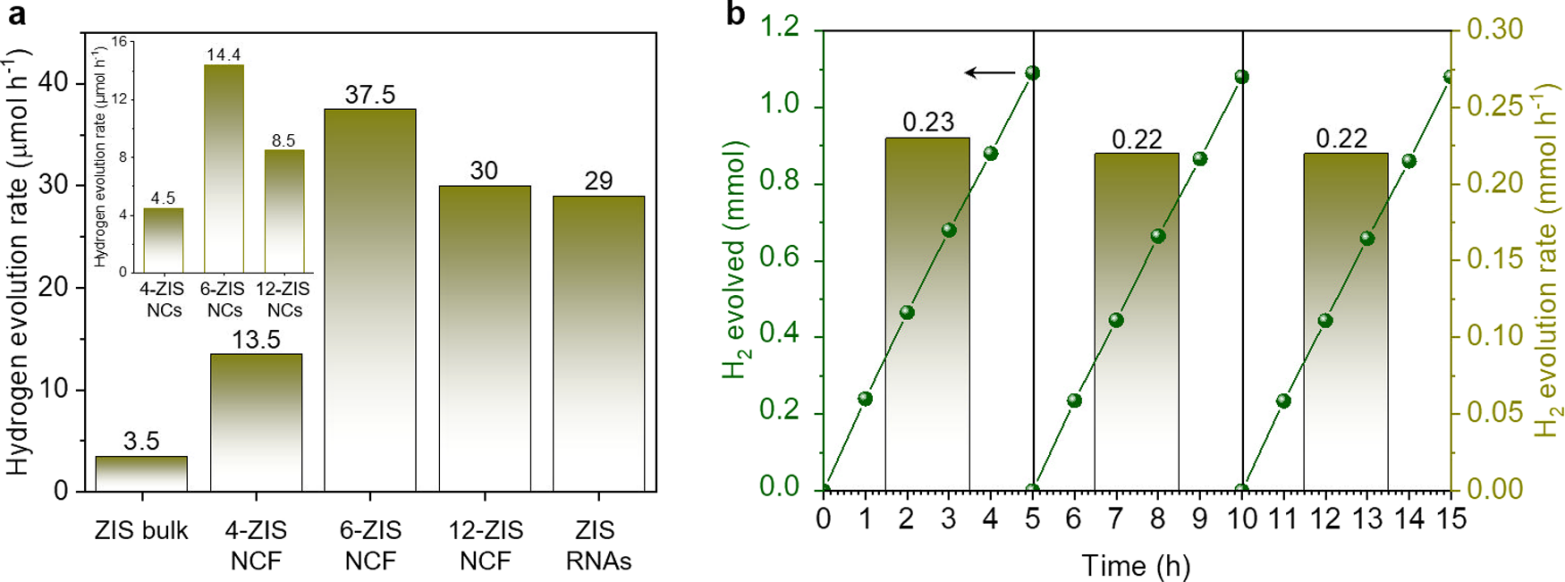
(a) Photocatalytic H_2_ generation rates of different ZIS catalysts under nonoptimized conditions (1 mg mL^–1^ catalyst in 0.35 M Na_2_S/0.25 M Na_2_SO_3_ aqueous solution; λ ≥ 380 nm light irradiation; 20 ± 2 °C). (b) Time-dependent hydrogen evolutions (lines) and average H_2_-production rates (column) at the course of the photocatalytic stability studies over 6-ZIS NCF catalyst. The H_2_-production rates were averaged over 5 h of illumination. The stability test was conducted with 1.5 mg mL^–1^ catalyst concentration in triethylamine (10% v/v) solution; 300 W Xe lamp irradiation (λ ≥ 380 nm).

The photocatalytic H_2_ evolution performances of the *n*-ZIS NCFs family were initially evaluated in a Na_2_S/Na_2_SO_3_-mixed solution using a custom-made gastight photocatalytic cell under λ ≥ 380 nm light irradiation. Figure 3a shows the photocatalytic H_2_ evolution activities of mesoporous ZIS samples along with that of isolated ZIS NCs, ZIS RNAs and polycrystalline ZIS sample. Although ZIS microparticles exhibit enhanced visible light absorption (bandgap energy ∼2.50 eV), they demonstrate limited hydrogen evolution activity (ca. 3.5 μmol h^–1^), which is primarily attributed to their low porosity and micrograin composition. Conversely, the open-pore structure and plethora of catalytic active sites of the mesoporous ZIS materials have an immediate impact on adsorption and photochemical reactions. Specifically, all *n*-ZIS NCFs samples exhibit a striking improvement in photocatalytic performance with a H_2_ evolution rate of 13.5 to 37.5 μmol h^–1^. The 6-ZIS NCF catalyst achieves the highest hydrogen production efficiency, which is nearly 10 times higher than that of the bulk counterpart, demonstrating a significant improvement in photocatalytic activity. As for the depressed activity observed for the mesoporous ZIS made of smaller (4 nm) NCs (∼13.5 μmol h^–1^), it is related to deficient charge-transfer kinetics prompted by the defective structure (see electrochemical results below). It is worth noting that the hydrogen production activity of 6-ZIS NCF also exceeds that of isolated ZIS NCs (∼4.5–14.4 μmol h^–1^) and ZIS RNAs (∼29.0 μmol h^–1^), which is obtained from direct coupling of colloidal 6 nm ZIS NCs, by a factor of 2.6–8.3× and 1.3×, respectively. These results unveil that both the small grain size of constituent nanoparticles and porous morphology are advantageous for enhancing photocatalytic H_2_-generation activity by providing shorter diffusion pathways for photogenerated carriers and a larger catalyst/liquid interface area. In control experiments, no hydrogen was evolved during the reaction in the dark or without a catalyst, indicating that the detected hydrogen originates from the photocatalytic water reduction reaction.

To further optimize the reaction conditions, we conducted a series of control experiments using different hole scavengers and catalyst loads. As depicted in Figure S12 in the Supporting Information, triethylamine (10% v/v) expedites the reaction kinetics of 6-ZIS NCF catalyst, resulting in an improved H_2_ evolution of 209 μmol h^–1^ compared to the 58 and 20–42 μmol h^–1^ H_2_ evolution rates observed under triethanolamine (10% v/v) and Na_2_S/Na_2_SO_3_ sacrificial conditions (at fixed catalyst mass), respectively. These results implicate the interfacial hole transfer for oxidation reaction as the rate-determining step for H_2_ evolution. Furthermore, the H_2_-evolution yield experiences a further increase with the catalyst concentration, reaching a maximum efficiency at 1.5 mg mL^–1^ (Figure S13, Supporting Information). Exceeding the above concentration, the photocatalytic performance slightly declines presumably due to light-scattering effects by the catalyst’s particles. Thus, upon optimization, 6-ZIS NCF attains an exceptional photocatalytic performance with a respective H_2_ evolution rate of 234 μmol h^–1^ (or 7.8 mmol g_cat_^–1^ h^–1^ mass activity) and apparent quantum yields (AQYs) of 25.0, 17.2, and 3.2% at 375, 420, and 440 ± 10 nm incident light wavelengths, respectively, assuming 100% absorption of the incident light. The wavelength-dependent AQY variation suggests that the light excitation of ZIS mesostructure is the driving force of the catalytic reaction. To our knowledge, this photocatalytic activity is among the highest reported thus far for thiospinel-based catalysts, and vastly higher than that of previously reported single sulfide photocatalysts. Other sulfide-based materials that exhibit such a high H_2_-evolution activity usually possess complex heterostructures of multi-ingredient composition. A comparison of the hydrogen evolution activity of our catalyst with previously reported catalysts is provided in Table S2 in the Supporting Information.

The stability of 6-ZIS NCF under photocatalytic conditions was examined through three 5 h catalytic tests. After each catalytic run, the photocatalyst was isolated from the reaction solution by centrifugation, washed several times with water, and redispersed in a fresh triethylamine solution. The hydrogen evolution tests reveal that 6-ZIS NCF maintained an exceptional photocorrosion resistance, showing no notable catalytic performance decay within 15 h operation (Figure 3b); 6-ZIS NCFs manifested an almost stable hydrogen release rate (ca. 0.22 mmol h^–1^), giving a total H_2_ generation amount of 3.25 mmol (∼78.2 mL) after 15 h of irradiation. Moreover, no obvious changes in chemical composition and oxidation states of the 6-ZIS NCF throughout catalysis were observed by EDS and XPS analyses (Figure S14, Supporting Information). Besides, the N_2_ physisorption isotherms of the reused catalyst demonstrate that the surface area (ca. 163 m^2^ g^–1^) and pore diameter (ca. 5.4 nm) undergo minimal changes after the prolonged photocatalysis test (Figure S15, Supporting Information). These results establish the excellent durability of the 6-ZIS NCF catalyst for the hydrogen evolution reaction.

### 2.3 Size-Dependent Electronic Properties of Mesoporous ZnIn_2_S_4_ Structures

**Figure 4:**
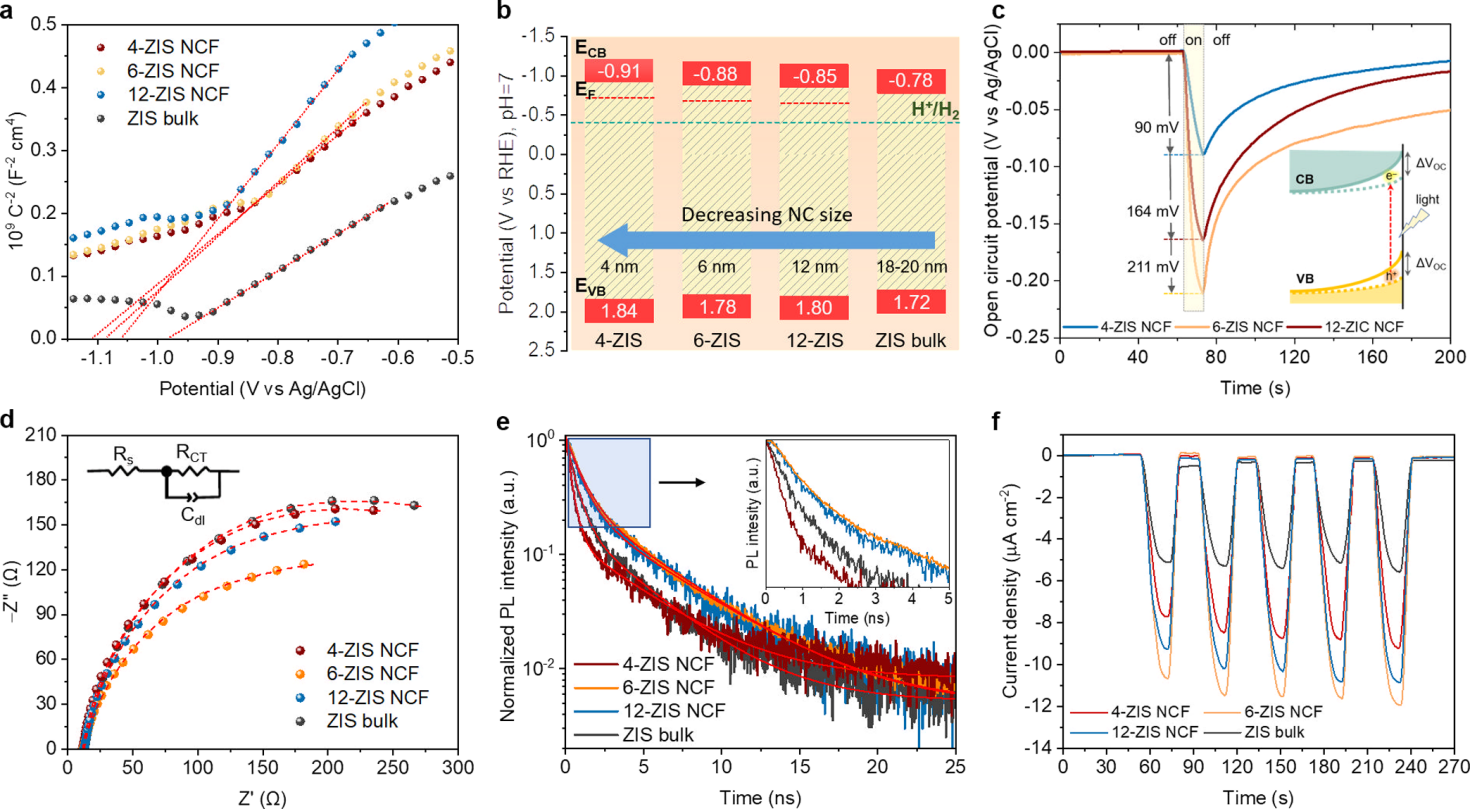
(a) Mott–Schottky plots and (b) energy band diagrams (*E*_CB_: conduction band energy, *E*_VB_: valence band energy, *E*_F_: Fermi level, H^+^/H_2_ redox potential) of different ZIS catalysts. (c) Open circuit potential versus elapsed time for mesoporous *n*-ZIS NCFs under switching on/off AM 1.5G illumination (10 s light on). The inset shows the change of the *V*_OC_ at the catalyst/liquid interface under chopped illumination; when the light is switched on, a charge accumulation occurs at the interface of ZIS NCs, mitigating the surface band bending. (d) EIS Nyquist plots (Inset: equivalent Randles circuit model), (e) time-resolved PL decay spectra under 375 nm laser pulse excitation (Inset: magnified view of the PL decay spectra) and (f) transient photocurrent spectra under the applied bias of −1 V (100 W visible-light-emitting diode) of the mesoporous *n*-ZIS NCFs and polycrystalline bulk ZIS catalysts. In panels (a, d, e), the red lines are fit to the experimental data.

To elucidate the size effect on the electronic structure of the *n*-ZIS NCFs materials, we performed electrochemical spectroscopy measurements in a 0.5 M Na_2_SO_4_ aqueous solution. Figure 4a shows Mott–Schottky plots, that is, the reciprocal square capacitance (1/*C*_SC_^2^) versus applied voltage (*E*), of different catalysts drop-casted as a thin film onto fluorine tin oxide (FTO, 10 Ω sq^–1^) electrodes. The flat-band potential (*E*_FB_) is determined by the intersection point of the linear segment in these plots, and all the measured potentials were converted to the reversible hydrogen electrode (RHE) scale at pH 7. All the 1/*C*_SC_^2^–*E* plots show positive slopes testifying ZIS samples to be n-type semiconductors, in accord with previous reports.^39,40^ By combining the *E*_FB_ potentials and the energy bandgaps (as obtained from UV–vis absorption spectra, Figure 2b), the energy levels of the valence band (*E*_VB_) for each catalyst can be calculated, and these data are included in Table S3 in the Supporting Information. In this analysis, *E*_FB_ serves as a good approximation of the CB edge position, which is quite feasible for heavily n-doped semiconductors (typically with >10^18^ cm^–3^ donor density),^41^ such as the ZIS. The above analysis shows that the band-edge positions of ZIS mesoporous vary systematically with the NC size. Compared with the polycrystalline ZIS (*E*_FB_ ∼ −0.78 V vs RHE), the mesoporous ensembles demonstrate a discernible increase in their *E*_FB_ potential on the energy scale, showing a shift from −0.85 V for the ∼12 nm-sized to −0.91 V (vs RHE) for the ∼4 nm-sized NC-consisting sample. The progressive cathodic shift of E_FB_ with decreasing NC size is consistent with the widening of the optical bandgap of *n*-ZIS NCFs as shown in Figure 2c, which is intrinsically induced by the quantum size effect of the constituent NCs. These effects are seen in Figure 4b, where the systematic variation of the band edges (*E*_FB_ and *E*_VB_) with NC size is illustrated. The shifts in the band-edge positions can be attributed to the limited number of electron wave functions contributing to the density of states in the conduction and valence bands as a result of the significant particle size reduction.^42,43^ Eventually, this leads to the discretization of energy levels between electronic states in the band structure, resulting in the widening of the bandgap.

The size-sensitive electronic structure of ZIS NCs aligns well with the results from density functional theory (DFT) calculations. Theoretical DFT studies have shown that ZnIn_2_S_4_ clusters exhibit a widening energy gap, increasing from 0.8 to 1.6 eV and further to 1.8 eV, as the lattice size contracts from 3 × 3 × 3, to 2 × 2 × 2, and to 1 × 1 × 1 unit cells (Figure S16, Supporting Information). Additionally, these ZnIn_2_S_4_ clusters demonstrate a transition from discrete localized electronic states to continuous bands with increasing lattice size, signifying improved electron conductivity. In line with quantum size effects, the two-dimensional ZnIn_2_S_4_ surface exhibits an energy bandgap of 0.7 eV. It should be noted that while DFT with the Perdew–Burke–Ernzerhof (PBE) approach tends to significantly underestimate the bandgap of crystalline semiconductors, it nonetheless accurately captures the overall electronic structure.^44^ We have verified this by repeating our calculations with the computationally expensive hybrid HSE06 functional, which confirmed a significantly more accurate bandgap (∼1.9 eV) while yielding a similar electronic density of states (DOS) for these compounds with both methods. In agreement with previous studies,^44^ the DOS profiles near the band edges of ZIS clusters reveal that the valence band is predominantly composed of S p orbitals, while the conduction band is primarily formed by In s and S p orbitals and a small contribution from Zn p orbitals. Consistent with theoretical calculations, the valence-band XPS (VB-XPS) spectra affirm a progressive upshift of the Fermi level (*E*_F_) with diminishing NC diameter. The results show that 4-ZIS NCF has the largest Fermi level offset from the VB maximum (2.61 eV), followed by 6-ZIS (2.51 eV) and then 12-ZIS (2.45 eV) NCFs (Figure S17, Supporting Information). This upshift in *E*_F_ position can be justified due to the sufficient elevation of the CB edge position via quantization of energy levels, in agreement with prior studies on individual metallic and semiconducting nanoparticles.^45^ In general, the elevation of the *E*_F_ energy level is advantageous for the efficient surficial electron transfer at the catalyst/liquid junction.^46^ Besides, an electron delocalization in the space charge region of *n*-ZIS NCFs is demonstrated by changes in charge carrier density (*N*_d_), as derived from the slope of the 1/*C*_sc_^2^–*E* lines, see Table S3 in the Supporting Information. When the ZIS NC size is reduced from 12 to 4 nm, the *N*_d_ of ZIS mesoporous undergoes a slight increase from ∼2.4 to ∼3.6 × 10^18^ cm^–3^, implying an increase in n-type doping. The trend in carrier concentration observed in the series of *n*-ZIS NCFs can be attributed to a reduced charge recombination rate, likely due to the narrow depletion layer width and resulting increased band bending within the ultrasmall nanoparticles. Although, the increase in free carriers due to the generation of sulfur vacancies (electron donors) in 4-ZIS NCF sample cannot be excluded. The depletion layer width (*W*_d_) calculated for ZIS mesoporous ranges from 13.6 to 11.5 nm (as shown in Table S3, Supporting Information) and is comparable to the size of ZIS NCs, contributing to a pronounced deformation of the band edges at the catalyst/liquid interface. This assumption is also supported by the results of the open-circuit voltage (*V*_OC_) measurements on *n*-ZIS NCFs during the photoexcitation process. In fact, the surface depletion field induced by band bending can be elucidated by the distinct variations in *V*_OC_ under steady-state and illumination conditions (Δ*V*_OC_ = *V*_OC,light_ – *V*_OC,dark_). As shown in Figure 4c, the light-induced *V*_OC_ shift (photovoltage) for ZIS mesoporous shows the trend: 6-ZIS (Δ*V*_OC_ = 211 mV) > 12-ZIS (Δ*V*_OC_ = 164 mV) > 4-ZIS (Δ*V*_OC_ = 90 mV). The higher photovoltage generated in 6-ZIS NCF suggests a sharper band bending, which generates a strong driving force (internal electric field) for the spatial dissociation of the photoexcited charge carrier, thereby amplifying the photocatalytic hydrogen evolution performance. The lower recombination rate of photogenerated carriers in this sample is further reaffirmed by the gradual decay of the *V*_OC_ signal over time after turning off illumination. Conversely, the diminished photovoltage amplitude and accelerated *V*_OC_ decay seen in 4-ZIS NCF are related to the surface pinning effect induced by midgap interface states (see below). Evidently, these findings collectively suggest that the substantial size reduction of constituent NCs dictates the electronic structure and, consequently, the photoredox activity of the catalysts. We conclude that quantization of the band-edge electronic states with the size decrease of ZIS NCs results in the elevation of the Fermi level and an increase of the band bending, which, in turn, accelerates charge separation within the depletion region and enhances interfacial charge transfer.

To provide more insights into the size-dependent kinetics of charge carrier recombination and transfer, the interfacial kinetics of the samples were explored with electrochemical impedance spectroscopy (EIS). Figure 4d shows the EIS Nyquist plots of the mesoporous and polycrystalline bulk ZIS materials (drop-cast on FTO electrodes) measured in 0.5 M Na_2_SO_4_ solution with a conventional three-electrode cell. The EIS data were fitted to an equivalent Randles circuit model, which consists of the electrolyte resistance (*R*_s_), double-layer capacitance (*C*_dl_) and charge-transfer resistance (*R*_ct_) (Figure 4d, inset). The above analysis discloses more efficient migration of electrons within the ZIS mesoporous frameworks (*R*_ct_ ∼ 307–377 Ω) compared to polycrystalline ZIS (395.1 Ω), accounting for the favorable carrier diffusion length in ZIS NCs. Remarkably, consistent with its superior photoactivity, 6-ZIS NCF exhibits a higher charge-transfer efficiency (307.0 Ω) across the catalyst/liquid interface, followed by 12-ZIS NCF (359.3 Ω) and 4-ZIS NCF (377.6 Ω), see Table S4 in the Supporting Information. Indeed, the decreasing trend of *R*_ct_ for *n*-ZIS NCFs aligns well with the results from hydrogen production shown in Figure 3a. To illustrate the effect of surface-active sites in the *n*-ZIS NCFs photocatalysts, we further conducted a comparative analysis of the double-layer capacitance (*C*_dl_) determined from the EIS data; the *C*_dl_ is directly proportional to the electrochemical active surface area (ECSA) at the catalyst/liquid interface. This analysis manifests an enhancement of the active surface area relative to the bulk ZIS of 1.2×, 2.8×, and 1.5× for the 4-ZIS, 6-ZIS, and 12-ZIS mesoporous structures, respectively, which accounts for the improved efficiency in interfacial charge transfer (Table S4, Supporting Information). Of note, 6-ZIS NCF outperforms all other samples in terms of ECSA, even surpassing the 4-ZIS NCF, despite the latter having a slightly larger specific surface area, meaning that 6-ZIS NCF has a higher intrinsic electrochemical activity. All this information explicitly demonstrates the positive effects of ZIS nanostructures in the transfer and separation efficiency of charge carriers and the exposure of catalytically active sites at the interface, which ultimately lead to enhanced photocatalytic performance.

The impact of constituent NCs on charge-carrier delocalization dynamics in mesoporous *n*-ZIS NCFs was further investigated by time-resolved photoluminescence (TRPL) spectroscopy and transient photocurrent (TPC) measurements. The PL decay spectra of the ZIS catalysts’ band-edge emission were analyzed using a biexponential function: *F*(*t*) = α_1_·e^–*t*/τ_1_^ + α_2_·e^–*t*/τ_2_^, where α_1_ and α_2_ are the relative amplitudes of each lifetime component, and τ_1_ and τ_2_ reflect the surface-mediated (fast) and intrinsic band-to-band (slow) relaxation of excited electrons, respectively (Figure 4e). Through this analysis, the weighted average lifetime (τ_av_) of 6-ZIS NCF is calculated to be 4.20 ns, much longer than the fluorescent lifetimes of mesoporous 4-ZIS (2.33 ns) and 12-ZIS (4.08 ns) NCFs as well as polycrystalline ZIS (3.20 ns), demonstrating its higher charge carrier separation capacity, in accordance with earlier *V*_OC_ results. Typically, in porous nanostructures with very small grain sizes (sub-10 nm), photoexcited electrons tend to preferentially transfer to the catalyst surface, actively participating in interface reaction rather than undergoing detrimental bulk recombination loss. The unexpectedly short-lived electron transfer (2.33 ns) in 4-ZIS NCF suggests the existence of additional pathways for fast electron–hole relaxations. This can be attributed to trap filling effect under illumination (that is induced by sulfur vacancies as evidenced by EDS and PDF analyses), which facilitates trapping of excited electrons and results in swift sub-band-to-VB transitions. Together with EIS results, this suggests that the presence of sulfur vacancies on the surface of 4-ZIS NCF diminishes electron accessibility at the catalyst interface by promoting the recombination of photoexcited carriers rather than their separation. This result is contrary to previous observations on sulfur-deficient ZnIn_2_S_4_ nanostructures.^29,47,48^ In line with this, a more cautious observation of the fast and slow decay time constants discloses an interesting correlation between the NCs size and τ-components of PL decay spectra. In mesoporous ZIS comprising smaller (ca. 4 nm) NCs the majority of the photoexcited electrons are lost via surface and/or defect-mediated recombination (α_1_ ∼ 83.3%) because of high defect trapping. On the contrary, as the NC size increases, the interface transitions associated with fast decay processes via surface defect states are overruled (α_1_ ∼ 36.4% for 6-ZIS and α_1_ ∼ 20% for 12-ZIS NCF), whereas band-edge relaxation emerges as the more prominent pathway for carrier recombination loss. The detailed information for TRPL fits is included in Table S5 in the Supporting Information. Consistent with the EIS findings, the efficient generation and migration of photoexcited carriers in ZIS mesoporous frameworks are further affirmed by TPC measurements. The current–potential (*J*–*V*) curves recorded under visible light (420–780 nm) illumination show that 6-ZIS NCF generates higher photocurrent (11.6 μA cm^–2^) than does the other mesoporous (8.4–10.2 μA cm^–2^) and polycrystalline (5.2 μA cm^–2^) ZIS materials (Figure 4f), corroborating the efficient separation and transfer of more photogenerated carriers to the interface. Taken together, all the above results consistently show that the photocurrent improvement and faster reaction kinetics of the ZIS mesoporous NC-frameworks primarily stem from enhanced dissociation and transfer of photoexcited electron–hole pairs. This enhancement is due to the low dimensionality of the constituent ZIS NCs, rather than the increased photon absorption or beneficial effect of surface trap states. Undoubtedly, these charge separation processes have significant implications for the hydrogen evolution reaction, as they substantially prolong the lifetime of the photogenerated charge carriers in the ZIS mesostructures, thus enabling more electrons and holes to participate in the electrochemical reactions at the catalyst’s surface.

## 3 Conclusions

In summary, we have developed a series of mesoporous frameworks derived from linked ZnIn_2_S_4_ nanocrystals of different sizes (ranging from ∼4 to ∼12 nm) by using a low-temperature colloidal synthetic route, followed by a polymer-templated self-assembly approach. These materials feature an open-pore nanostructure with a large BET surface area (up to 207 m^2^ g^–1^) and exhibit strong size-dependent electronic and catalytic properties. The effect of nanocrystal size on band-edge positions and charge-transfer kinetics is thoroughly investigated by a combination of spectroscopic and electrochemical methods. The characterization results suggest that the size reduction of ZIS nanocrystals brings high interfacial charge-transfer kinetics and charge separation rates, thereby amplifying the ability of photogenerated carriers to initiate water-splitting reactions. For ultrasmall nanocrystals, the kinetics of charge transfer and separation rates further confirm the dominant effects of the surface sulfur vacancies on carrier recombination losses, which are fundamental reasons for depressed photocurrent and thus reduced photocatalytic activity. Benefiting from the short diffusion path of charge carriers, high donor density and suppressed charge recombination, the mesoporous ensembles made of 6 nm-sized ZIS NCs demonstrate a remarkable photocatalytic hydrogen evolution performance, yielding a 7.8 mmol h^–1^ g_cat_^–1^ H_2_-evolution rate and a photon-to-hydrogen conversion efficiency of 25.0% at 375 nm and 17.2% at 420 nm. The above findings highlight the importance of the rational design of photocatalysts and provide fundamental insights into the charge-transfer dynamics and electronic properties of nanocatalysts for clean energy conversion reactions. Moreover, the open-pore architecture of ZIS nanostructures offers opportunities for surface chemical modification, thereby expanding the synthesis of advanced multicomponent semiconductors with improved interfacial properties. We foresee intriguing electronic characteristics within these systems, as well as potential applications in photocatalysis.

## 4 Materials and Methods

### 4.1 Synthesis of ZIS NCs

Size-controlled ZnIn_2_S_4_ nanocrystals were synthesized according to a previously reported synthetic protocol.^25^ In a typical reaction, Zn(NO_3_)_2_·6H_2_O (1 mmol), In(NO_3_)_2_·6H_2_O (2 mmol), 3-mercaptopropionic acid (3-MPA, 24 mmol) and NH_4_OH (25 wt %, 12 mL) were dissolved in 20 mL of ethylene glycol (C_2_H_6_O_2_). The mixture was heated to 150 °C under reflux conditions and then an ethylene glycol (10 mL) solution of thioacetamide (10 mmol) was injected, forming a pale-yellow colloidal suspension. To obtain ZnIn_2_S_4_ nanocrystals of different sizes, the reaction time was adjusted from 3 to 6 and to 12 h to yield nanoparticles with size of ∼4, ∼6, and ∼12 nm (denoted as ZIS NCs), respectively. After the solution reached room temperature, the ZnIn_2_S_4_ nanocrystals were isolated through the addition of isopropyl alcohol, washed several times with deionized (DI) water/ethanol (1:1 v/v) mixture, and then dried in air at 40 °C for 24 h.

For comparison, polycrystalline bulk ZnIn_2_S_4_ (denoted as ZIS bulk) was also prepared using a common hydrothermal method. In a typical procedure, stoichiometric amounts of Zn(NO_3_)_2_·6H_2_O (1 mmol) and In(NO_3_)_2_·6H_2_O (2 mmol), and an excessive amount of thioacetamide (10 mmol) were mixed with 20 mL of DI water in a 30 mL Teflon-line autoclave and subsequently heated to 150 °C for 12 h. The obtained yellow product was isolated through centrifugation, washed with water and ethanol, and finally dried at 60 °C for 24 h.

### 4.2 Synthesis of Mesoporous ZIS NCFs

Mesoporous frameworks of ZnIn_2_S_4_ nanocrystals (denoted as *n*-ZIS NCFs, where *n* refers to the particle size of ZnIn_2_S_4_ nanocrystals) were obtained as follows: 250 mg of 3-MPA-capped ZnIn_2_S_4_ nanocrystals was suspended in 2.5 mL of DI water forming a homogeneous solution.

For the formation of a stable colloidal dispersion, a small amount (a few drops) of 10 M NH_4_OH was added. The dispersion was then added into an aqueous solution containing the polymer template (Pluronic Brij-58) (2.5 mL, 10% w/v) and kept under vigorous stirring for 1 h at room temperature. Subsequently, 1.2 mL of H_2_O_2_ (3% v/v) was added dropwise to the above solution until a gel suspension was formed. The gel-like solution was left to evaporate the solvent at 40 °C for 3 days under static conditions. The removal of the organic template was achieved by washing twice with warm ethanol (ca. 40 °C) for 2 h and then three times with DI water for 1 h each. The final product was isolated through filtration, washed several times with ethanol and DI water, and finally dried at 60 °C for 24 h. Also, random aggregates of 6 nm-sized ZnIn_2_S_4_ nanocrystals (denoted as ZIS RNAs) were prepared using a similar procedure but without the presence of the organic template.

### 4.3 Physical Characterization

A PANalytical X’pert Pro MPD X-ray diffractometer equipped with Cu Ka radiation (λ = 1.5418 Å) was used to examine the crystallinity of the prepared samples. Small-angle X-ray scattering (SAXS) patterns were performed on a Xeuss 3.0 (Xenocs, France) system equipped with a two-dimensional (2D) detector and a Cu rotating anode (λ = 1.5418 Å). TGA patterns were collected on a Discovery TGA5500 (TA Instruments) under N_2_ flow at ∼200 mL min^–1^ and a heating rate of 10 °C min^–1^. Field-emission SEM (FESEM) images and EDS spectra were acquired using a JEOL JSM-IT700HR scanning electron microscope, operating at 20 kV. An accumulation time of 60 s and at least ten different regions of each sample were employed for data acquisition. A JEOL JEM-2100 electron microscope with a LaB_6_ filament at an acceleration voltage of 200 keV was employed to derive the TEM images. The samples were suspended in an ethanol solution and then were drop-casted on a carbon-coated Cu grid. X-ray photoelectron spectroscopy was performed on a SPECs spectrometer equipped with a Phoibos 100 1D-DLD analyzer and Al Kα radiation source (1486.6 eV). The reported binding energies were corrected in regards to the C 1s adventitious carbon signal (284.8 eV). UV–vis/near-IR diffuse reflectance data were collected on a Shimadzu UV-2600 spectrophotometer. BaSO_4_ was used as a 100% reflectance standard and the diffuse reflectance data were converted to absorbance using the Kubelka–Munk function: α/*S* = (1 – *R*)^2^/(2*R*), in which *R* is the measured reflectance and α, *S* are the absorption and scattering coefficients, respectively. N_2_ physisorption experiments were performed on a Quantachrome NOVA 3200*e* analyzer at −196 °C. Before measurement, each sample was degassed at 100 °C under vacuum conditions (<10^–5^ Torr) for 12 h. The specific surface areas were calculated by applying the Brunauer–Emmet–Teller (BET) method on the adsorption data in relative pressure (*P*/*P*_0_) range of 0.04–0.24.^49^ The total pore volumes were calculated at *P*/*P*_0_ = 0.98, while the pore-size distribution plots were obtained using nonlocal density functional theory (NLDFT) model on the adsorption isotherms.^50^ Time-resolved photoluminescence (TRPL) measurements were performed on an Edinburgh FS5 spectrofluorometer. TRPL spectra were acquired at room temperature using 375 nm pulse laser excitation. High-energy X-ray diffuse scattering (HE-XRDS) plots were collected on a Bruker D8 Venture diffractometer equipped with a PHOTON II CPAD detector at room temperature, using Mo Kα radiation (λ = 0.7093 Å) under capillary geometry. Diffraction data were corrected for the empty cell scattering. The X-ray total scattering data were Fourier transformed to obtain the PDFs using PDFgetX3.^51^

### 4.4 Electrochemical Measurements

The electrochemical experiments were conducted on a single-channel VersaSTAT 4 electrochemical workstation (Princeton Applied Research) with a three-electrode configuration. The electrochemical cell contains a working electrode, an Ag/AgCl (sat. KCl) as reference electrode and a Pt wire as counter electrode. For the preparation of the working electrodes, 10 mg of the sample was dispersed in 1 mL of ethanol under ultrasonic treatment for 1 h. Then, 500 μL of the dispersion was drop-casted on a fluorine-doped tin oxide (FTO, 10 Ω sp^–1^) glass and dried at 60 °C for 1 h. Mott–Schottky plots were recorded in a 0.5 M Na_2_SO_4_ aqueous solution (pH = 6.8) at a frequency of 1 kHz and a voltage amplitude of 10 mV AC. All measured potentials were converted to reversible hydrogen electrode (RHE) using the following equation
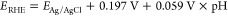
1where, *E*_Ag/AgCl_ is the measured potential using an Ag/AgCl reference electrode.

The donor density (*N*_d_) of the samples was calculated from the Mott–Schottky plots using the equation
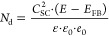
2where, *C*_SC_ is the space-charge capacitance, *E* is the applied voltage, *E*_FB_ is the flat-band potential, ε is the dielectric constant of ZnIn_2_S_4_ (4.73),^52^ ε_0_ is the dielectric permittivity in vacuum (8.8542 × 10^–12^ C V^–1^ m^–1^), and *e*_0_ is the elementary charge (1.602 × 10^–19^ C).

The width of the depletion layer (*W*_d_) was calculated as
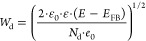
3Electrochemical impedance spectroscopy (EIS) measurements were carried out in a frequency range of 1 Hz to 10 kHz with an applied voltage of −1.2 V (vs Ag/AgCl) and an amplitude of 10 mV. The EIS data were fitted to an equivalent electrical circuit using ZView software. Transient photocurrents were obtained in 0.5 M Na_2_SO_4_ electrolyte using a bias potential at −1 V (vs Ag/AgCl) under chopped visible light (420–780 nm) illumination. Open circuit potential measurements were performed in a 0.5 M Na_2_SO_4_ solution under switching on/off sunlight (AM 1.5G) irradiation. The light was illuminated through the FTO side (back-side illumination).

### 4.5 Theoretical Calculations

First-principles calculations were performed within density functional theory (DFT) using the Vienna Ab Initio Software Package (VASP)^53^ with the projector-augmented wave (PAW) method for core electrons and nuclei,^54^ the generalized gradient approximation (GGA) of Perdew–Burke–Ernzerhof (PBE) for exchange–correlation functional,^55^ and a plane-wave basis with cutoff energy of 500 eV. Energy was converged to 10^–5^ eV and the Monkhorst–Pack mesh for the first Brillouin zone sampling was adapted to different geometries. The simulation unit cell of hexagonal ZnIn_2_S_4_ was taken from JCPDS card no. 65–2023 and the structures presented here are those of the infinite monolayer and a series of clusters with increasing size, that is, 1 × 1 × 1, 2 × 2 × 1, and 3 × 3 × 1 repeating unit cells. Atomic positions were fully relaxed and converged to 0.01 eV Å^–2^. Periodic boundary conditions were applied to the monolayer with 15 Å of vacuum in the direction perpendicular to the surface. A mesh of 5 × 5 × 1 *k*-points was used, which became 15 × 15 × 1 for the calculation of the electronic density of states. The clusters were surrounded by a vacuum of 15 Å in all directions, and calculations were performed with a single *k*-point.

### 4.6 Photocatalytic Measurements

The photocatalytic hydrogen evolution experiments were performed in a custom-built airtight Pyrex photocatalytic reactor. Before light irradiation, 20 mg of the catalyst was dispersed in 20 mL of aqueous solution containing 0.35 M Na_2_S·9H_2_O and 0.25 M Na_2_SO_3_ (or other sacrificial agent) and the dispersion was degassed using argon gas for at least 30 min to remove the presence of atmospheric air. Then, the photocatalytic reactor was inserted in a water-cooling system to maintain a constant temperature (20 ± 2 °C) and was irradiated with a 300 W Xe lamp (Variac Cermax). The hydrogen evolution was analyzed by a gas chromatograph (Shimadzu GC-2014) equipped with a thermal conductivity detector. The apparent quantum yield (AQY) was assessed through the quantification of the hydrogen evolution at 375, 420, and 440 ± 10 nm monochromatic light irradiation, using the following equation
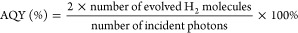
4The photon intensity of the incident light at 375 nm (9.68 mW cm^–2^), 420 nm (11.71 mW cm^–2^) and 440 nm (20.37 mW cm^–2^) was measured using a StarLite power meter with a FL400A-BB-50 thermal detector (Ophir Optronics Ltd.).

## Supporting Information Available

Elemental composition, EDS spectra, PL lifetime, and electrochemical data of *n*-ZIS NCFs and polycrystalline ZIS samples; photocatalytic H_2_-production activity data of different thiospinel-based catalysts; TGA profiles, XPS spectra, VB-XPS spectra, SAXS patterns and particle size distribution plots of *n*-ZIS NCFs; XRD pattern and FESEM images of polycrystalline ZIS; TEM images and N_2_ physisorption isotherms for mesoporous 4-ZIS and 12-ZIS NCFs samples; UV–vis spectrum of ZIS RNAs; catalytic data, XPS spectra, and N_2_ physisorption isotherms of 6-ZIS NCF catalyst, and DFT results of different ZnIn_2_S_4_ clusters (PDF)
