# Supplementary material for: Size Dependent Photocatalytic Activity of Mesoporous ZnIn2S4 Nanocrystal Networks
Source: ACS Catal. 2024 Sep 12;14(18):14251–62. doi: 10.1021/acscatal.4c04195 (PMC11420945; doi:10.1021/acscatal.4c04195)
Supplement: Supplementary file 1 — cs4c04195_si_001.pdf [file cs4c04195_si_001.pdf]

Size Dependent Photocatalytic Activity of Mesoporous $ZnIn_2S_4$ Nanocrystal Networks

*Evangelos K. Andreou†, Ioannis Vamvasakis†, Andreas Douloumis†, Georgios Kopidakis†, Gerasimos S. Armatas†,**

†Department of Materials Science and Engineering, University of Crete, Heraklion 70013, Greece

*To whom correspondence should be addressed, E-mail: garmatas@materials.uoc.gr

Supporting Tables

Table S1. Elemental composition of the mesoporous *n*-ZIS NCFs and polycrystalline ZIS materials.

Sample	Zn (at.%)	In (at.%)	S (at.%)	Zn:In:S[a]
4-ZIS NCF	16.2	28.4	55.4	1.14:2.00:3.90
6-ZIS NCF	14.7	27.6	57.7	1.06:2.00:4.14
12-ZIS NCF	14.4	27.2	58.4	1.05:2.00:4.25
ZIS bulk	14.1	28.5	57.4	0.99:2.00:4.03

[a]EDS Zn/In/S atomic ratio based on In atoms.

Table S2. Photocatalytic efficiency comparison of several thiospinel-based photocatalysts.

Photocatalyst	Reaction conditions	Light source	H_2 evolution rate		QE	Ref.
			(μmol h^{-1})	(μmol g^{-1} h^{-1})		
$CdIn_2S_4/ZnS$	20 mg catalyst, 0.35 M Na_2S, 0.25 M Na_2SO_3	300 W Xe lamp	74.8	3743	2.2% at 365 nm	[1]
$CdIn_2S_4/ZnIn_2S_4$	4 mg catalyst, 0.35 M Na_2S, 0.25 M Na_2SO_3	300 W Xe lamp (λ≥420 nm)	50.7	12670	18.7% at 420 nm	[2]
Pt@$ZnIn_2S_4$	200 mg catalyst, 0.35 M Na_2S, 0.25 M Na_2SO_3	300 W Xe lamp	122.2	611	11.9 % at 420 nm	[3]
Pt@$ZnIn_2$S4	100 mg catalyst, 0.35 M Na_2S, 0.25 M Na_2SO_3	300 W Xe lamp	220	2200	13.16 % at 420 nm	[4]
$ZnIn_2S_4$	3 mg catalyst, 20 % v/v TEOA	300 W Xe lamp	5.8	1940	10.1 % at 420 nm	[5]
Pt@Cu-dopped $ZnIn_2S_4$	200 mg catalyst, 0.35 M Na_2S, 0.25 M Na_2SO_3	300 W Xe lamp	151.5	758	9.6 % at 420 nm	[6]
N-doped $ZnIn_2S_4$	20 mg catalyst, 10 % v/v TEOA	300 W Xe lamp	236	11806	16.1 % at 420 nm	[7]
1 wt.% MoS_2/ $CdIn_2S_4$	50 mg catalyst, 0.35 M Na_2S, 0.25 M Na_2SO_3	300 W Xe lamp (λ≥420 nm)	47.3	2365	5.2% at 400 nm	[8]
$CdIn_2S_4$/$In(OH)_3$/Ni Cr-LDH	50 mg catalyst, 0.35 M Na_2S, 0.25 M Na_2SO_3	300 W Xe lamp (λ≥420 nm)	54.65	1093	1.7% at 420 nm	[9]
$Ni_2P/ZnIn_2S_4$	50 mg catalyst, 10% v/v lactic acid	300 W Xe lamp (λ>400 nm)	103.3	2066	7.7% at 420 nm	[10]
CoP@$ZnIn_2S_4$	10 mg catalyst, 10% v/v TEOA	300 W Xe lamp (λ≥420 nm	103	10300	16.2% at 420 nm	[11]
**6-ZIS NCF**	**30 mg catalyst, 10% v/v TEA**	**300 W Xe lamp**	**234**	**7800**	**25.0% at 375 nm, 17.2% at 420 nm**	**This work**

Table S3. Electrochemical properties, determined through EIS analysis of the mesoporous *n*-ZIS NCFs and polycrystalline ZIS materials.

Sample	E_{FB}	E_{VB}	Donor density (N_d, cm^{-3})	Depletion layer width (W_d, nm)
	(V vs RHE, pH 7)			
4-ZIS NCF	–0.91	1.84	3.58 x 10^{18}	11.5
6-ZIS NCF	–0.88	1.78	3.32 x 10^{18}	11.8
12-ZIS NCF	–0.85	1.80	2.41 x 10^{18}	13.6
ZIS bulk	–0.78	1.72	4.85 x 10^{18}	9.2

Table S4. Nyquist equivalent circuit fitted parameters of the mesoporous *n*-ZIS NCFs and polycrystalline ZIS materials.

**Sample**	**R_s (Ω)**	**C_{dl} (F cm^{-2})**	**ECSA[a]**	**R_{ct} (Ω)**	**x^2**
4-ZIS NCF	11.6	75.1 x 10^{-6}	1.2	377.6	1.8 x 10^{-4}
6-ZIS NCF	11.8	180.0 x 10^{-6}	2.8	307.0	2.2 x 10^{-4}
12-ZIS NCF	12.4	96.8 x 10^{-6}	1.5	359.3	1.7 x 10^{-4}
ZIS bulk	12.9	64.6 x 10^{-6}	1	395.1	1.1 x 10^{-4}

[a]Electrochemically active surface area (ECSA) increment obtained as a ratio of the double-layer capacitance relative to that of bulk ZIS.

Table S5. Time-resolved photoluminescence decay parameters of the mesoporous *n*-ZIS NCFs and polycrystalline ZIS materials.

**Sample**	**τ_1 (ns)**	**τ_2 (ns)**	**α_1 (%)**	**α_2 (%)**	**τ_{av}[a] (ns)**
4-ZIS NCF	0.60	3.73	83.3	16.7	2.33
6-ZIS NCF	0.90	4.57	36.4	63.6	4.20
12-ZIS NCF	0.37	4.17	20.0	80.0	4.08
ZIS bulk	0.86	4.75	23.2	76.8	3.29

[a]The average lifetime (τ_{av}) was determined using the following equation: $\tau_{av} = (\sum_i \alpha_i \tau_i^2)/(\sum_i \alpha_i \tau_i)$ (i = 1, 2).

Supporting Figures

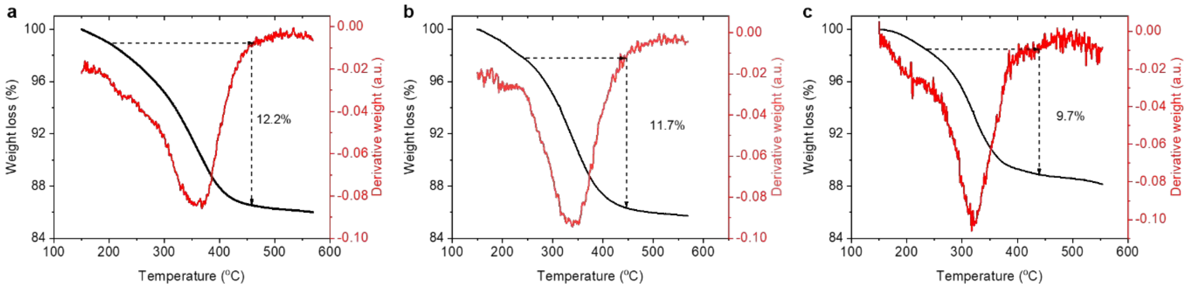

Figure S1. TGA profiles (black lines) and the corresponding differential thermogravimetric (DTG) curve (red line) of (a) 4-ZIS, (b) 6-ZIS and (c) 12-ZIS NCFs, depicting a weight loss of □9.7–12.2% at a temperature range of 230 to 460 °C due to the decomposition of remaining organic residue.

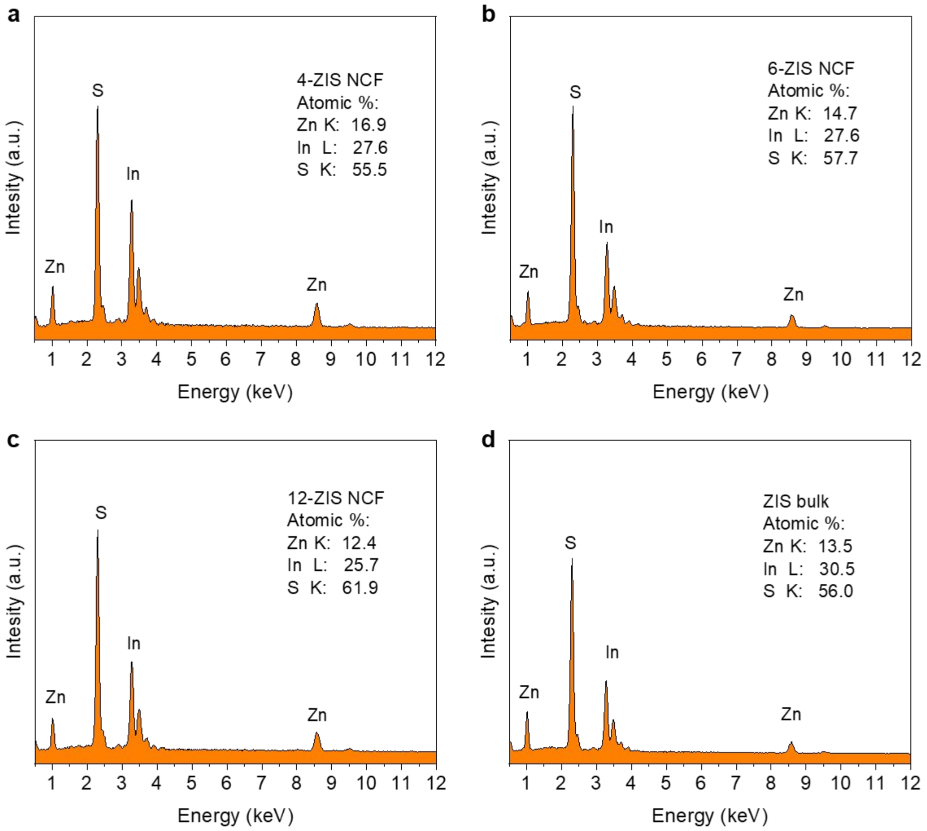

Figure S2. Typical EDS spectra of the mesoporous *n*-ZIS NCFs and bulk polycrystalline ZIS.

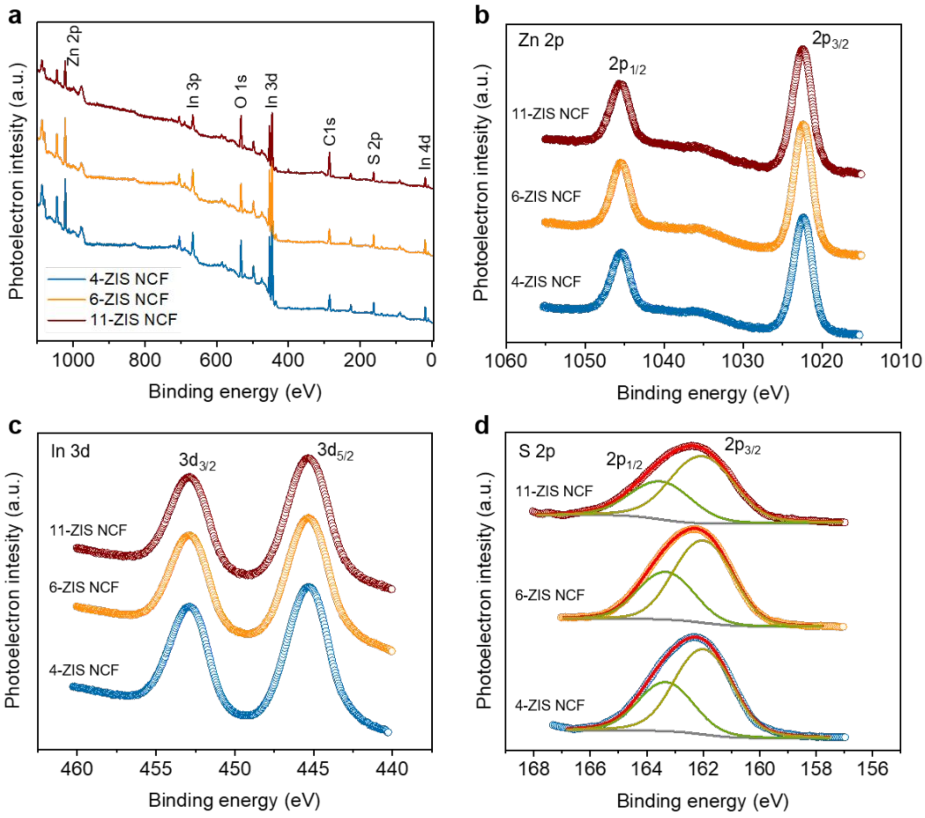

Figure S3. (a) XPS survey scans and high-resolution XPS core-levels of (b) Zn 2p, (c) In 3d and (d) S 2p of the mesoporous *n*-ZIS NCFs. In panel d: the XPS S 2p deconvoluted spectra of S $2p_{3/2}$ and S $2p_{1/2}$ core-levels are represented as yellow and green curves. The red lines are fits to the experimental data.

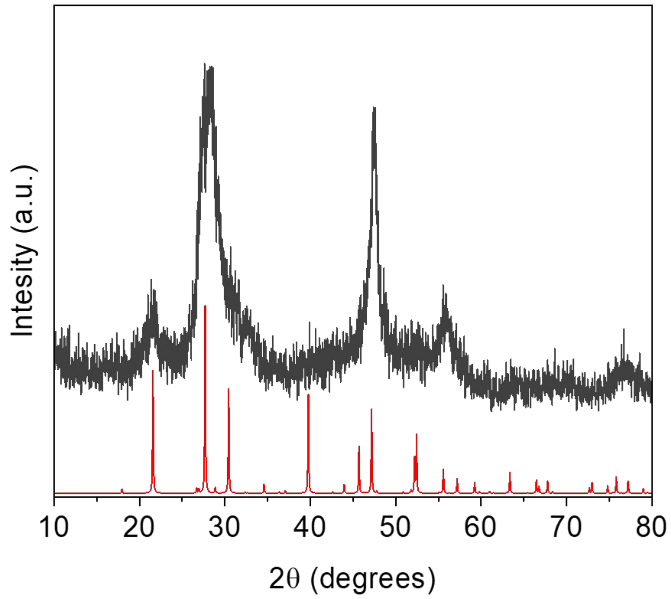

Figure S4. XRD pattern of the bulk-like $ZnIn_2S_4$ catalyst. The red lines correspond to the diffraction peaks of the hexagonal $ZnIn_2S_4$ (*P*-3*m*1, JCPDS card no. 65-2023).

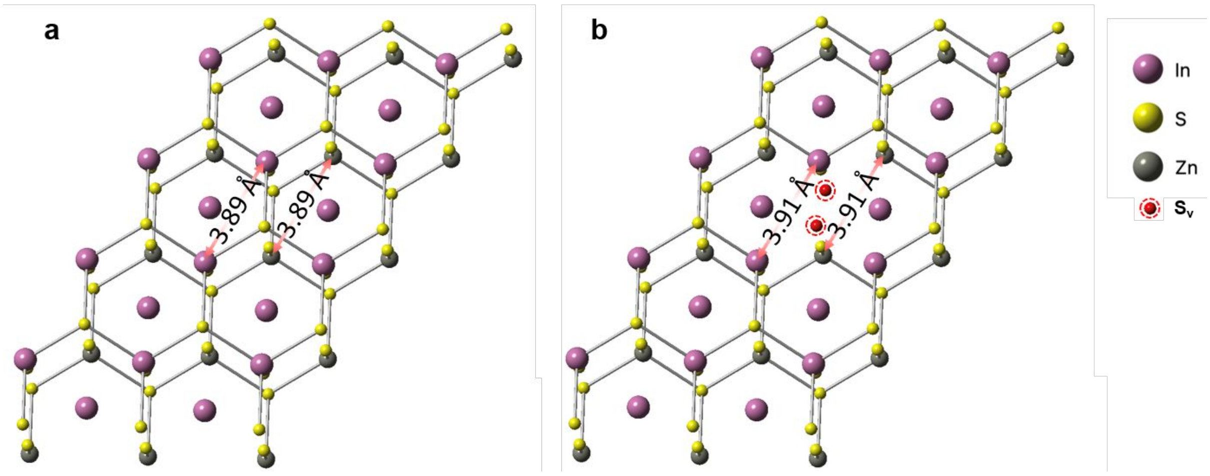

Figure S5. The In···In and Zn···Zn interatomic distances in the hexagonal lattice of ZIS (a) without and (b) with sulfur vacancies (S_v). The presence of sulfur vacancies in the lattice of ZIS induces bond dislocations and thus alters the arrangement (interatomic distance) of neighboring Zn and In atoms.

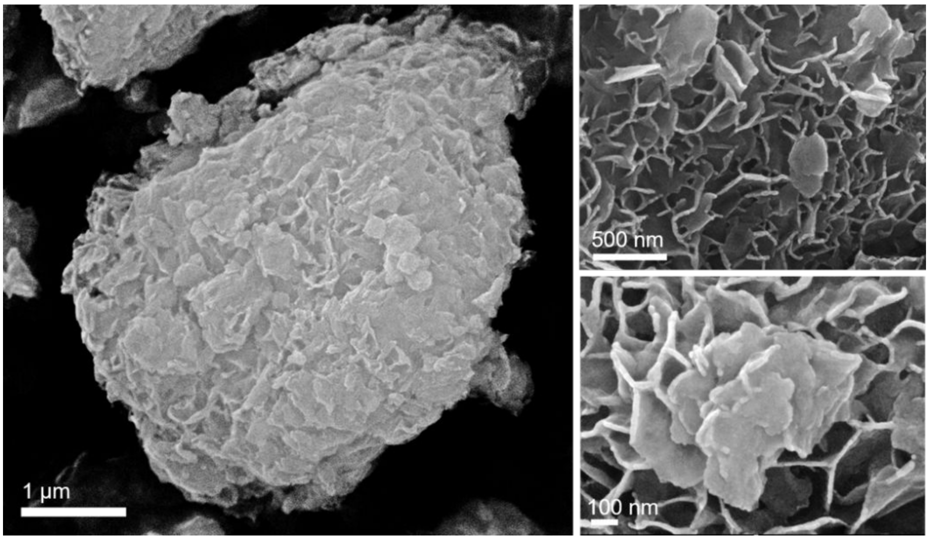

Figure S6. Typical FESEM images of the bulk polycrystalline ZIS, showing micro-sized particles of 3–5 µm diameter. The bulk ZIS particles exhibit a flowerlike morphology composing by plenty of intersecting nanosheets with thickness of ~18–20 nm.

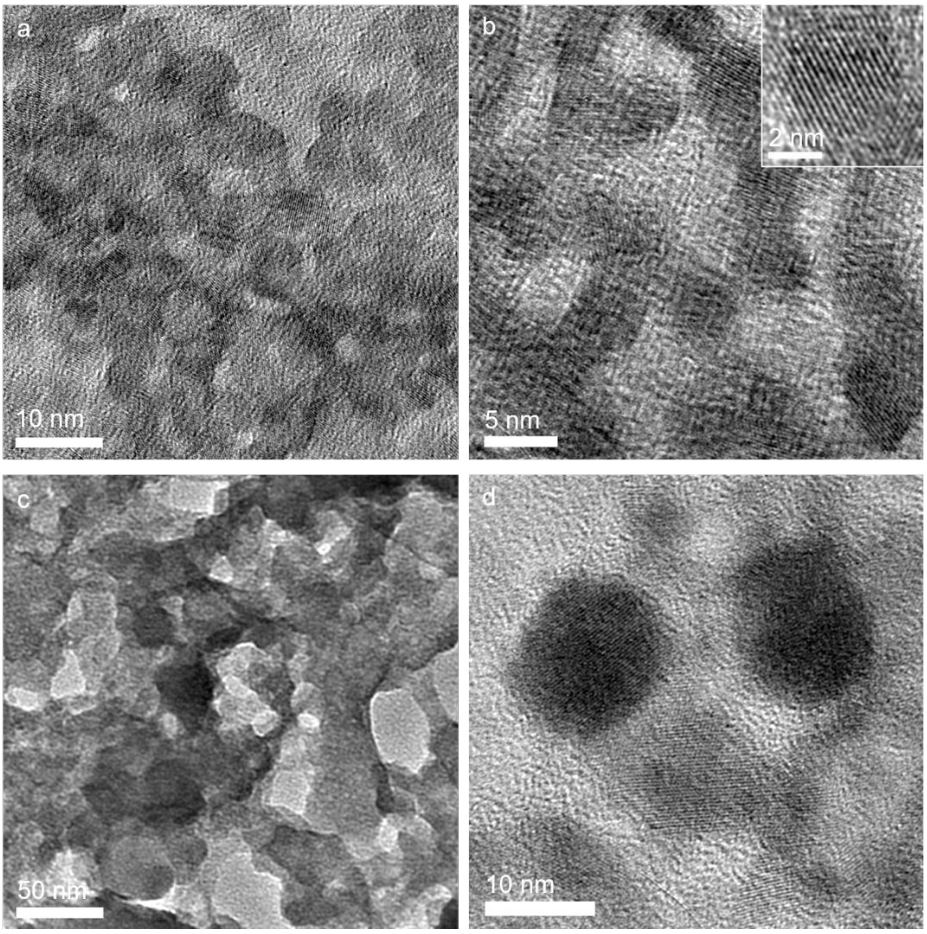

Figure S7. Typical TEM images of the mesoporous (a-b) 4-ZIS and (c-d) 12-ZIS NCFs.

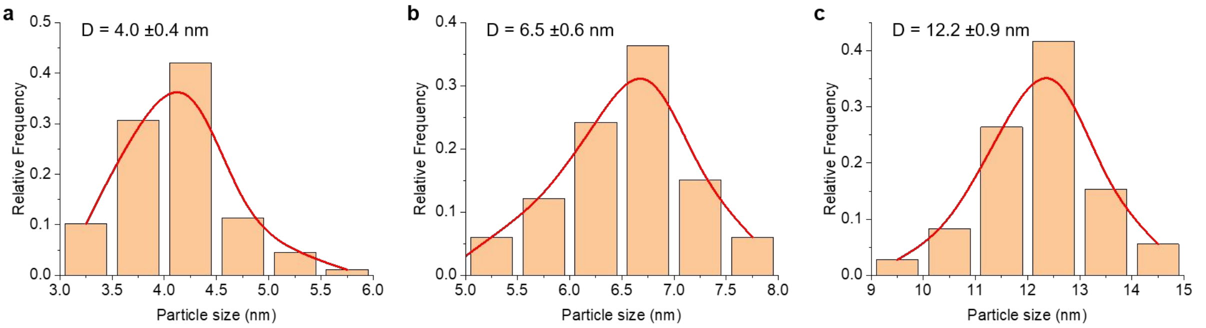

Figure S8. Histograms of the size distributions of ZIS NCs for mesoporous (a) 4-ZIS, (b) 6-ZIS and (c) 12-ZIS NCFs. Histograms were obtained based on a count of more than 50 NCs.

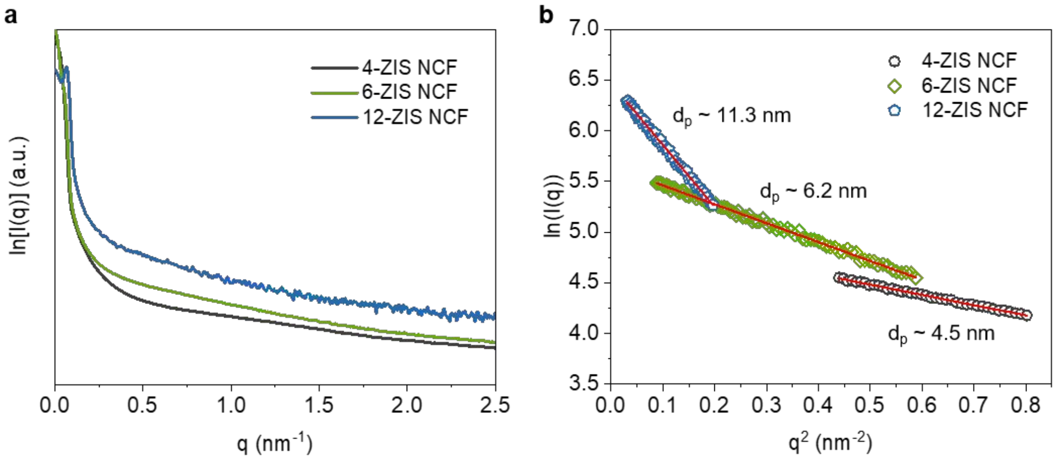

Figure S9. (a) SAXS pattens and (b) Guinier plots [I(q) ∝ $-q^2R_g^2/3$, where q is the scattering vector and R_g is the radius of gyration] of the mesoporous n-ZIS NCFs, yielding an average particle size (d_p) from ~4.5 to ~11.3 nm.

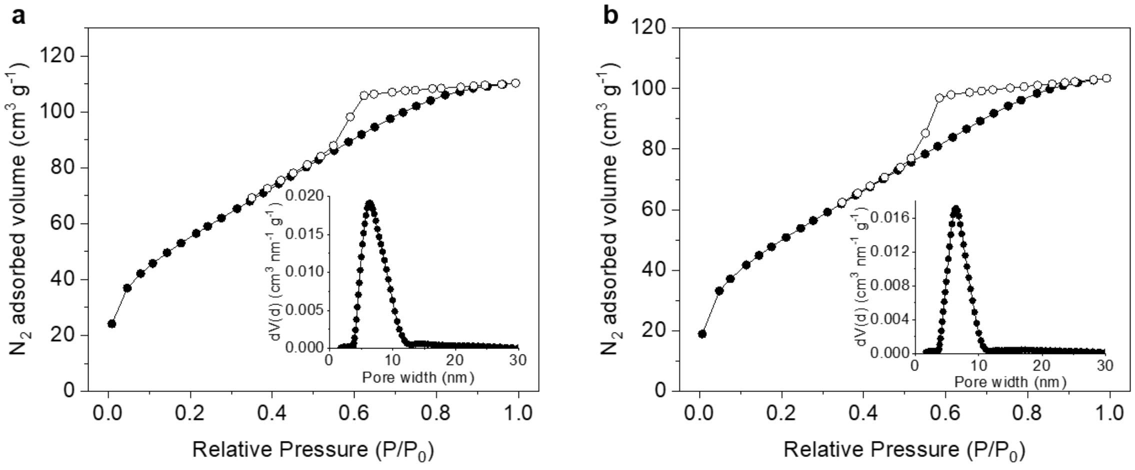

Figure S10. N_2 adsorption (filled symbols) and desorption (empty symbols) isotherms at –196 °C for the mesoporous (a) 4-ZIS and (b) 12-ZIS NCFs. Inset: the corresponding NLDFT pore-size distribution plot derived from the adsorption data.

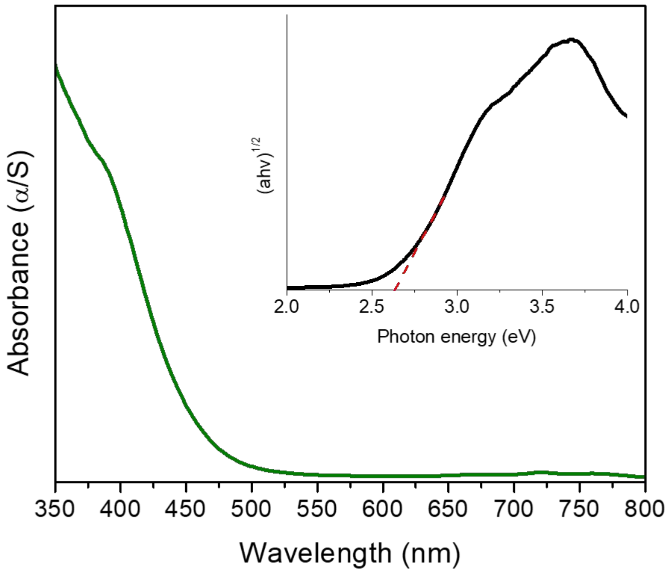

Figure S11. UV-vis absorption spectrum and the corresponding Tauc plot (inset) of random ZIS NC-aggregates (ZIS RNAs).

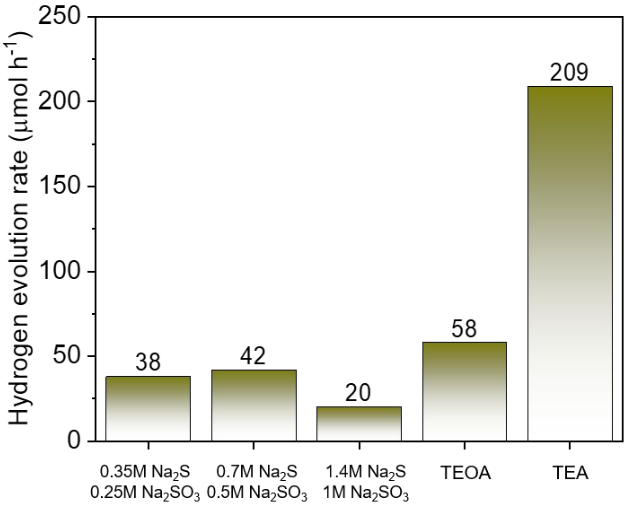

Figure S12. Photocatalytic hydrogen evolution activity of the mesoporous 6-ZIS NCF catalyst using different sacrificial reagents: 0.35-1.4M Na_2S/0.25-1M Na_2SO_3 mixed solution, 10% v/v triethanolamine (TEOA) and 10% v/v triethylamine (TEA). All the photocatalytic experiments were conducted at a fixed catalyst concentration (1 mg mL^{-1}), under UV-vis ($\lambda \geq 380$ nm) irradiation (300-W Xe lamp) at 20 ±2 ^{o}C.

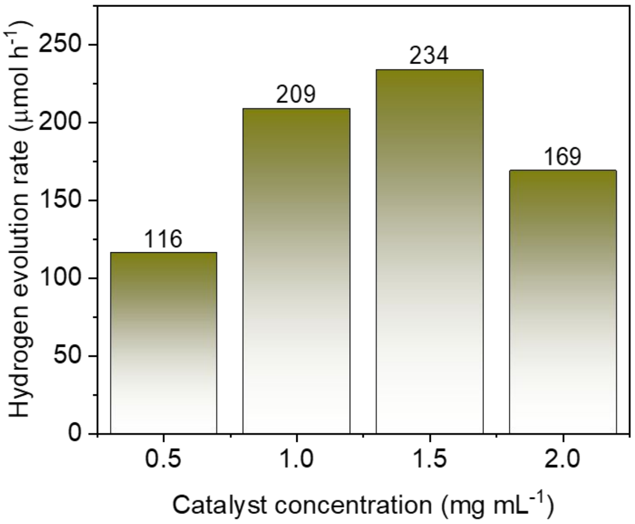

Figure S13. Photocatalytic H_2 evolution activities for different loadings of 6-ZIS NCF catalyst. Experimental conditions: 0.5–2 mg mL^{-1} of catalyst concentrations, 20 mL aqueous solution containing 10% (v/v) TEA; UV-vis ($\lambda \geq 380$ nm) irradiation (300-W Xe lamp), 20 ±2 °C.

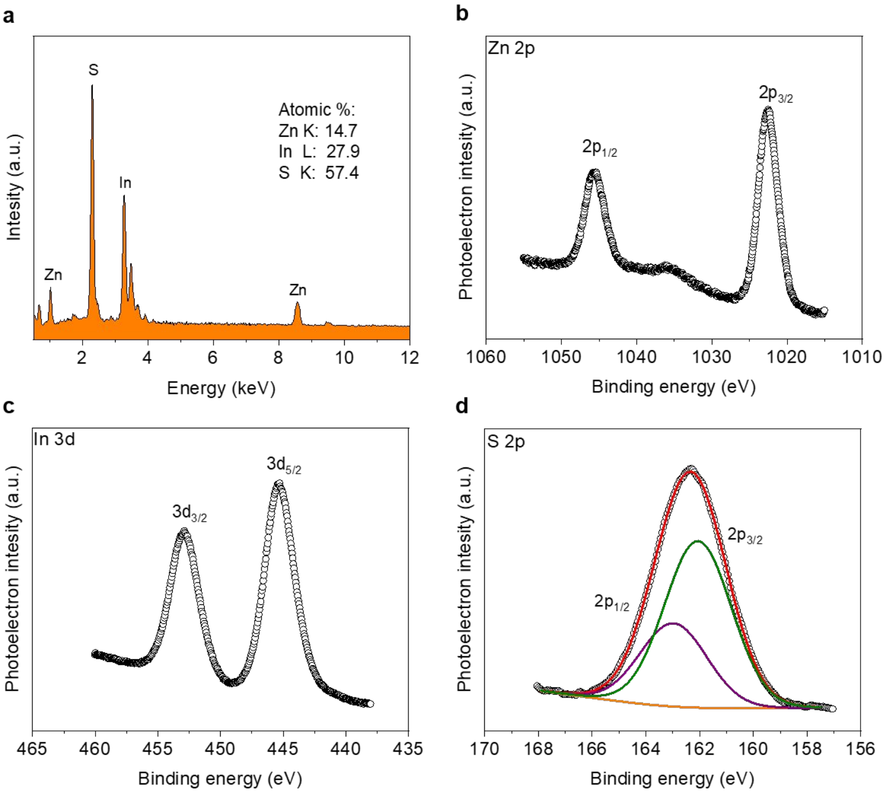

Figure S14. (a) Typical EDS spectrum and XPS core-level spectra of the (a) Zn 2p, (b) In 3d and (c) S 2p of the reused 6-ZIS NCF catalyst. EDS analysis indicates Zn/In/S atomic rations ~1:1.9:3.9, very close to the composition of the fresh sample.

The Zn 2p XPS spectrum of the recycled 6-ZIS NCF catalyst shows a doublet peak at 1022.4 and 1045.5 eV binding energies, corresponding to the Zn $2p_{3/2}$ and Zn $2p_{1/2}$ core-levels of divalent Zn ions, respectively. In the In 3d region, the peaks at 445.4 and 452.9 eV binding energies are attributed to the In $3d_{5/2}$ and In $3d_{3/2}$ core-levels of In^{3+} oxidation state. The S 2p XPS spectrum shows two deconvoluted peaks at 163.0 and 162.1 eV, associated with the $S2p_{3/2}$ and S $2p_{1/2}$ core-levels of the S^{2-} valence state.

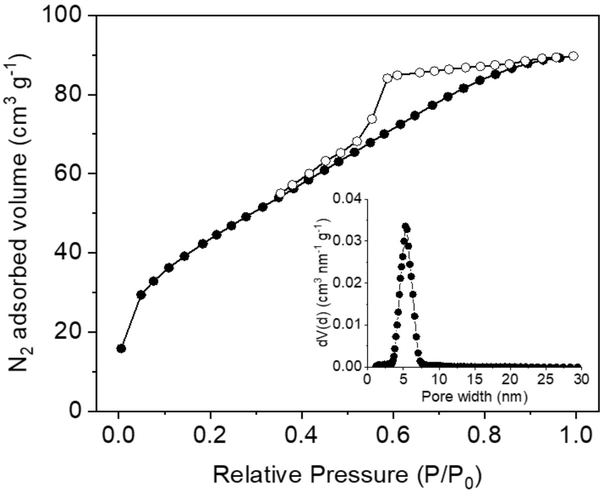

Figure S15. N_2 adsorption-desorption isotherms at –196 °C and the corresponding NLDFT pore size distribution plot (inset) of the 6-ZIS NCF catalyst retrieved after 15 hours of photocatalytic reaction. Analysis of the adsorption data with the BET method gives surface area of 163 $m^2\ g^{-1}$, total pore volume of 0.15 $cm^3\ g^{-1}$, and pore size of 5.4 nm.

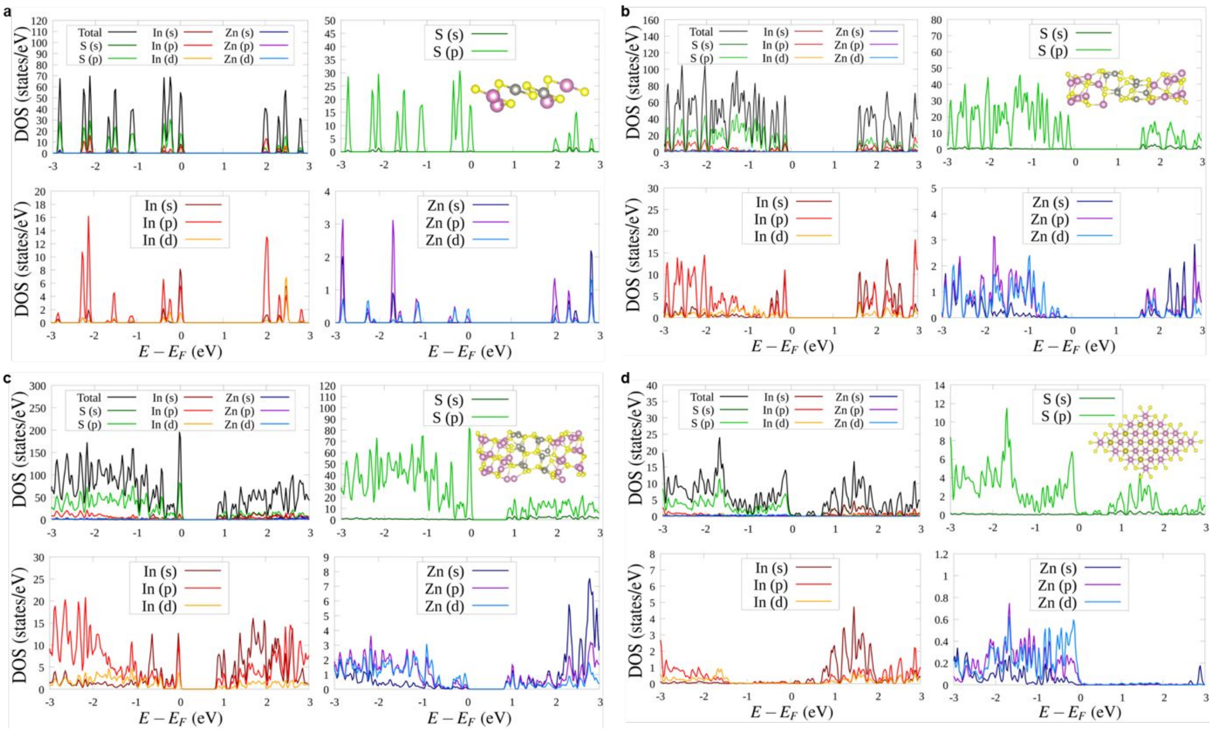

Figure S16. Total density-of-state (DOS) and isolated orbital-projected DOS profiles for (a) 1×1×1, (b) 2×2×2 and (c) 3×3×3 unit-cells and (d) two-dimensional $ZnIn_2S_4$ calculated using GGA potential.

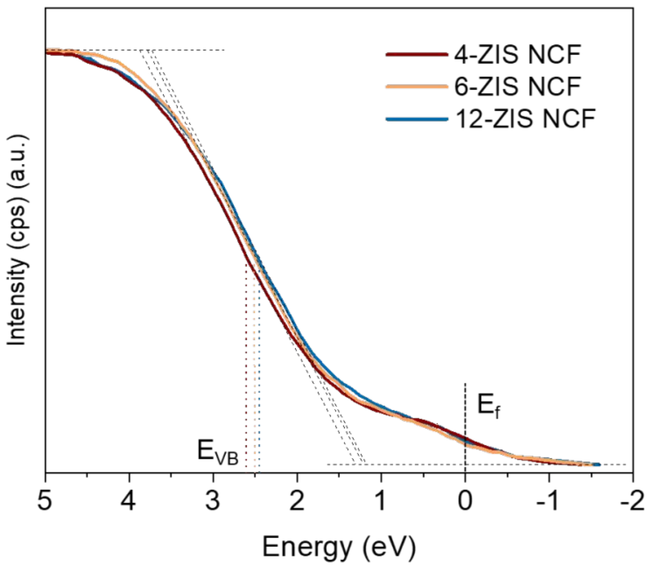

Figure S17. VB-XPS spectra of the mesoporous *n*-ZIS NCFs. The half maximum of the step height of the VB spectra dictates a Fermi level offset ($E_{VB} – E_f$) of 2.61, 2.51 and 2.45 eV for the 4-ZIS, 6-ZIS and 12-ZIS NCFs, respectively.

References

(1) Xie, L.; Liu, G.; Suo, R.; Xie, Z.; Liu, H.; Chen, J.; Chen, J.; Lu, C.-Z. Construction of a Z-Scheme $CdIn_2S_4/ZnS$ Heterojunction for the Enhanced Photocatalytic Hydrogen Evolution. *J. Alloys Compd.* **2023**, *948*, 169692.

(2) Dang, X.; Xie, M.; Dai, F.; Guo, J.; Liu, J.; Lu, X. The in Situ Construction of $ZnIn_2S_4/CdIn_2S_4$ 2D/3D Nano Hetero-Structure for an Enhanced Visible-Light-Driven Hydrogen Production. *J. Mater. Chem. A* **2021**, *9*, 14888–14896.

(3) Shen, S.; Zhao, L.; Guo, L. Crystallite, Optical and Photocatalytic Properties of Visible-Light-Driven $ZnIn_2S_4$ Photocatalysts Synthesized via a Surfactant-Assisted Hydrothermal Method. *Mater. Res. Bull.* **2009**, *44*, 100–105.

(4) Shen, J.; Zai, J.; Yuan, Y.; Qian, X. 3D Hierarchical $ZnIn_2S_4$: The Preparation and Photocatalytic Properties on Water Splitting. *Int. J. Hydrogen Energy* **2012**, *37*, 16986–16993.

(5) Shi, X.; Mao, L.; Yang, P.; Zheng, H.; Fujitsuka, M.; Zhang, J.; Majima, T. Ultrathin $ZnIn_2S_4$ Nanosheets with Active (110) Facet Exposure and Efficient Charge Separation for Cocatalyst Free Photocatalytic Hydrogen Evolution. *Appl. Catal. B* **2020**, *265*, 118616.

(6) Shen, S.; Zhao, L.; Zhou, Z.; Guo, L. Enhanced Photocatalytic Hydrogen Evolution over Cu-Doped $ZnIn_2S_4$ under Visible Light Irradiation. *J. Phys. Chem. C* **2008**, *112*, 16148–16155.

(7) Du, C.; Yan, B.; Lin, Z.; Yang, G. Enhanced Carrier Separation and Increased Electron Density in 2D Heavily N-Doped $ZnIn_2S_4$ for Photocatalytic Hydrogen Production. *J. Mater. Chem. A* **2019**, *8*, 207–217.

(8) Prabhu, Y. T.; Kumari, R.; Gautam, A.; Sreedhar, B.; Pal, U. Highly Oriented MoS_2@$CdIn_2S_4$ Nanostructures for Efficient Solar Fuel Generation. *Nano-Structures & Nano-Objects* **2021**, *26*, 100682.

(9) Fu, R.; Gong, Y.; Li, C.; Niu, L.; Liu, X. $CdIn_2S_4/In(OH)_3$/NiCr-LDH Multi-Interface Heterostructure Photocatalyst for Enhanced Photocatalytic H_2 Evolution and Cr(VI) Reduction. *Nanomaterials* **2021**, *11*, 3122.

(10) Li, X. Li; Wang, X. Jing; Zhu, J. Yu; Li, Y. Pei; Zhao, J.; Li, F. Tang. Fabrication of Two-Dimensional $Ni_2P/ZnIn_2S_4$ Heterostructures for Enhanced Photocatalytic Hydrogen Evolution. *Chem. Eng. J.* **2018**, *353*, 15–24.

(11) Wu, B.; Liu, N.; Lu, L.; Zhang, R.; Zhang, R.; Shi, W.; Cheng, P. A MOF-Derived Hierarchical CoP@$ZnIn_2S_4$ Photocatalyst for Visible Light-Driven Hydrogen Evolution. *Chem. Comm.* **2022**, *58*, 6622–6625.
